# Smoothened receptor signaling regulates the developmental shift of GABA polarity in rat somatosensory cortex

**DOI:** 10.1242/jcs.247700

**Published:** 2020-10-23

**Authors:** Quentin Delmotte, Mira Hamze, Igor Medina, Emmanuelle Buhler, Jinwei Zhang, Yesser H. Belgacem, Christophe Porcher

**Affiliations:** 1 Aix-Marseille University, Parc Scientifique de Luminy, 13273, Marseille, France; 2 INSERM (Institut National de la Santé et de la Recherche Médicale) Unité 1249, Marseille, Parc Scientifique de Luminy, 13273 Marseille, France; 3 INMED (Institut de Neurobiologie de la Méditerranée), Parc Scientifique de Luminy, 13273 Marseille, France; 4 Plateforme Post-Génomique, INMED, 13273 Marseille, France; 5 Institute of Biomedical and Clinical Sciences, Medical School, College of Medicine and Health, University of Exeter, Hatherly Laboratories, Exeter EX4 4PS, UK

**Keywords:** Smoothened receptor, Sonic hedgehog, KCC2, Chloride homeostasis, GABAergic transmission

## Abstract

Sonic hedgehog (Shh) and its patched–smoothened receptor complex control a variety of functions in the developing central nervous system, such as neural cell proliferation and differentiation. Recently, Shh signaling components have been found to be expressed at the synaptic level in the postnatal brain, suggesting a potential role in the regulation of synaptic transmission. Using *in utero* electroporation of constitutively active and negative-phenotype forms of the Shh signal transducer smoothened (Smo), we studied the role of Smo signaling in the development and maturation of GABAergic transmission in the somatosensory cortex. Our results show that enhancing Smo activity during development accelerates the shift from depolarizing to hyperpolarizing GABA in a manner dependent on functional expression of potassium–chloride cotransporter type 2 (KCC2, also known as SLC12A5). On the other hand, blocking Smo activity maintains the GABA response in a depolarizing state in mature cortical neurons, resulting in altered chloride homeostasis and increased seizure susceptibility. This study reveals unexpected functions of Smo signaling in the regulation of chloride homeostasis, through control of KCC2 cell-surface stability, and the timing of the GABA excitatory-to-inhibitory shift in brain maturation.

## Abstract (precis)

**Summary:** The smoothened receptor controls the time course of inhibitory transmission by regulating stability of the potassium-chloride cotransporter KCC2 at the plasma membrane.

## INTRODUCTION

Sonic hedgehog (Shh) is an activity-dependent secreted synaptic molecule (Beug et al., 2011). In the presence of Shh ligand, the receptor patched-1 (Ptch1) relieves its constitutive inhibition of the G-protein-coupled transducer smoothened (Smo), leading to activation of downstream signaling factors (Belgacem and Borodinsky, 2015; Briscoe and Thérond, 2013; Riobo et al., 2006). Shh signaling plays a wide range of functions during embryonic development from neuronal cell proliferation to differentiation (Belgacem et al., 2016; Briscoe and Thérond, 2013; Ruat et al., 2012; Traiffort et al., 2010). Furthermore, in the developing mammalian central nervous system, Shh signaling is involved in axonal elongation (Hammond et al., 2009; Parra and Zou, 2010; Yao et al., 2015) and formation of the cortical connectivity (Harwell et al., 2012; Memi et al., 2018). After birth, Shh signaling components are found in several brain regions, including the cerebral cortex and hippocampus, from an early stage of postnatal development to adulthood (Charytoniuk et al., 2002; Petralia et al., 2011, 2012; Rivell et al., 2019; Traiffort et al., 2010). In the adult hippocampus, the Shh pathway plays an essential role in neurogenesis in the dentate gyrus (Antonelli et al., 2019; Breunig et al., 2008). In addition, Shh receptor Ptch1 and its signal transducer Smo are also expressed at the synaptic junctions of the immature and adult hippocampus (Charytoniuk et al., 2002; Mitchell et al., 2012; Petralia et al., 2011), suggesting roles other than control of neurogenesis (Zuñiga and Stoeckli, 2017). For instance, Shh signaling was reported to exert a modulatory action on neuronal electrical activity in the adult brain (Bezard et al., 2003; Pascual et al., 2005) and, more recently, Shh–Smo signaling has been shown to regulate the formation of glutamatergic and GABAergic terminals in hippocampal neurons (Mitchell et al., 2012). These morphological changes are accompanied by an increase in the frequency of excitatory postsynaptic currents (Feng et al., 2016; Mitchell et al., 2012). Taken together, these findings support the view that the Shh signaling pathway plays a crucial role during early postnatal neuronal circuit construction and synaptic plasticity in the hippocampus and cortex. Thus, its impairment may affect neuronal network formation and lead to brain dysfunction. Likewise, both in human and animal models, accumulating evidence indicates that impairment of the Shh–Smo pathway at postnatal stages may contribute to the emergence of neurodevelopmental disorders, including autism spectrum disorders (ASD) (Al-Ayadhi, 2012; Halepoto et al., 2015) and seizures (Feng et al., 2016; Su et al., 2017).

In the early postnatal life, GABA_A_ receptor function changes from depolarizing to hyperpolarizing in a KCC2 (also known as SLC12A5)-dependent manner (Ben-Ari et al., 1989; Rivera et al., 1999). Alteration in the GABA developmental sequence leads to a compromised balance between excitatory and inhibitory transmission that is associated with severe changes in the neuronal network circuitry (Ben-Ari, 2002; Ben-Ari et al., 2007; Kilb et al., 2013; Sernagor et al., 2010; Wang and Kriegstein, 2009) and, subsequently, with the onset of neurodevelopmental brain disorders (Ben-Ari and Holmes, 2005; Kuzirian and Paradis, 2011; Mueller et al., 2015).

Although both Shh–Smo and GABA signaling are critically involved in similar processes of neuronal network formation and are implicated in etiology of the same neurodevelopmental disorders, their putative interplay remains unclear. Here we postulated that Shh components may contribute to the onset of the GABA polarity shift and suggested that deregulation of the Shh–Smo signaling pathway might then lead to an impaired neuronal network and to the emergence of brain disorders in adulthood. Because loss of Smo function results in an embryonic lethal phenotype (Zhang et al., 2001) and Smo activation in Wnt1-Cre;R26SmoM2 mice induces a hyperplasia of the facial processes at embryonic stages (Jeong et al., 2004), we used *in utero* electroporation (IUE) to target the somatosensory cortex with constructs encoding either negative-phenotype (Smo Δ570–581, referred to as Smo-ΔN) (Kim et al., 2009) or constitutively active (Smo A1, referred to as Smo-CA) (Chen et al., 2011) Smo variants. Our results reveal an unexpected function for the Smo signaling pathway in regulation of GABAergic developmental timing in the rat cerebral cortex, notably through the modulation of cell-surface expression and stability of KCC2. We further show that impairment of Smo signaling alters the phosphorylation state of KCC2, thus maintaining a depolarizing action of GABA, which leads to an alteration of GABAergic inhibitory transmission and might contribute to the emergence of brain disorders.

## RESULTS

### Cortical expression pattern of Smo-CA and Smo-ΔN

**Fig. 1.**
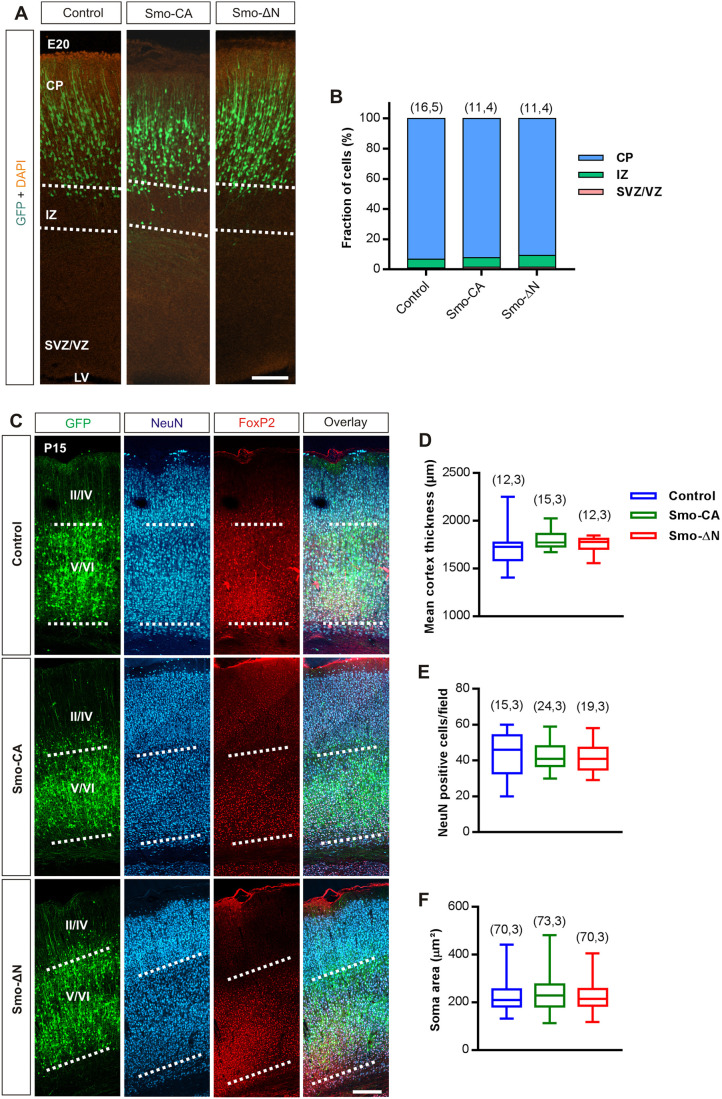
**Cortical expression pattern of Smo-related constructs.** (A) Representative distribution of electroporated neurons in rat developing cortex at E20 with GFP alone (Control) or GFP with Smo-constitutively active (Smo-CA) or Smo negative-phenotype (Smo-ΔN) mutants. CP, cortical plate; IZ, intermediate zone; LV, lateral ventricle; SVZ/VZ, subventricular/ventricular zone. Dotted lines indicate boundaries between zones. Nuclei are stained with DAPI. Scale bar: 30 µm. (B) Quantification of cortical distribution of electroporated neurons between CP (blue), IZ (green) and SVZ/VZ (red). (C) Representative neocortical section showing laminar position of electroporated cells processed postnatally at P15 and co-immunostained with NeuN (blue) and FoxP2 (red). GFP fluorescence intensity profile shows that E15-generated neurons are distributed mainly in deeper layers V/VI. Dotted lines indicate the boundaries between layers. Scale bar: 250 µm. (D) Box plots of cortical thickness and (E) neuronal density in GFP- (control), Smo-CA- and Smo-ΔN-expressing brains. (F) Box plots of neuron soma size in electroporated rats at P15. Box plots show the interquartile range with the median indicated. Whiskers show the range. Number of slices and rats are indicated in parenthesis.

Previous studies have shown that Shh signaling pathway components, including Ptch1 and Smo, are present in the postnatal mammalian brain (Harwell et al., 2012; Mitchell et al., 2012; Petralia et al., 2011; Rivell et al., 2019). In the mouse neocortex, Shh signaling regulates the formation of neuronal networks in immature brain, suggesting a potential function in construction and maturation of neural circuits during the postnatal periods. In order to evaluate the role of Shh signaling in the developing brain, we used IUE of plasmids to express GFP alone, as a control, or in combination with constitutively active (Smo-CA) or negative-phenotype (Smo-ΔN) forms of Smo, an essential component for Shh signaling. IUE of rat brains was performed at embryonic day 15 (E15) in order to target mainly pyramidal neurons of layers V and VI (Kriegstein and Noctor, 2004). Because Shh signaling is involved in cell division and growth of cortical progenitors (Araújo et al., 2014; Radonjić et al., 2016), we investigated whether Smo-related constructs might produce alterations in the localization of electroporated neuronal cells in embryonic day 20 (E20) rat embryos. We found no significant difference between the distribution of cells electroporated with Smo-CA or Smo-ΔN and the distribution of GFP-only control cells (Fig. 1A,B) within the cortical plate (CP) (mean of 92.28%, 90.79% and 93.34%, respectively; *P*=0.37 for Smo-CA and *P*=0.26 for Smo-ΔN, Mann–Whitney test), the intermediate zone (IZ) (6.10%, 7.71% and 5.62%, respectively; *P*=0.74 for Smo-CA and *P*=0.21 for Smo-ΔN, Mann–Whitney test) and the subventricular/ventricular zones (SVZ/VZ) (1.61%, 1.49% and 1.04%, respectively; *P*=0.58 for Smo-CA and *P*=0.49 for Smo-ΔN, Mann–Whitney test). These findings suggest that electroporation of Smo-related constructs has no gross impact at the cellular level on maturation and localization of electroporated cells in developing rat somatosensory cortex.

We next performed immunostaining of electroporated tissues at postnatal day 15 (P15) using the neuronal markers NeuN and FoxP2 (deeper layer neuron markers) to assist in the histological identification of cortical layers (Fig. 1C). The fluorescence intensity profile of both Smo-CA- and Smo-ΔN-expressing neurons did not show any obvious differences in the cortical layering when compared to that of control neurons. Thus, Smo-CA- or Smo-ΔN-expressing neurons electroporated at E15 most likely represent layers V and VI of the somatosensory cortex. The cortical thickness of Smo-CA- and Smo-ΔN-expressing rats measured at P15 was not different from that of control GFP-expressing rats (median of 1772 µm, 1779 µm and 1772 µm, respectively; *P*=0.07 for Smo-CA and *P*=0.17 for Smo-ΔN, Mann–Whitney test; Fig. 1C,D). The density of NeuN immuno-positive cells in Smo-CA and Smo-ΔN electroporated somatosensory cortices, measured in layer V, revealed no difference when compared to that in control tissues [46 neurons per observation field for control versus 41 neurons for Smo-CA (*P*=0.72) and 41 neurons for Smo-ΔN (*P=*0.44), Mann–Whitney test; Fig. 1E]. Similarly, the average soma neuronal size remained unchanged (210.3 µm² for control versus 229.2 μm² for Smo-CA and 213.8 µm² for Smo-ΔN; *P*=0.26 and *P*=0.48 when compared to control, respectively; Mann–Whitney test; Fig. 1F).

Taken together, these observations indicate that expression of Smo-CA or Smo-ΔN does not alter the mean neuronal density or the positioning of pyramidal neurons in the cerebral cortex.

### Smo-related constructs control presynaptic terminal density but not apoptosis

**Fig. 2.**
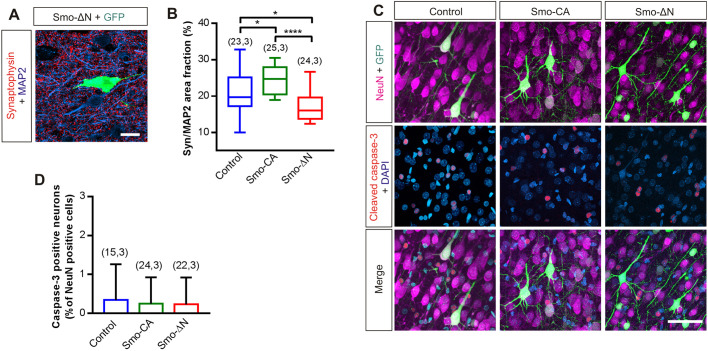
**Smo-related constructs control presynaptic terminal density but not apoptosis.** (A) Example of immunofluorescence signal of a P30 cortical section expressing Smo-ΔN and GFP (green), co-immunostained with synaptophysin (red) and MAP2 (blue). Scale bar: 30 µm. (B) Box plots of synaptophysin area fraction normalized to MAP2 area fraction. Box plots show the interquartile range with the median indicated. Whiskers show the range. (C) Immunofluorescence signal of P15 cortical sections electroporated at E15 with GFP (control) with or without Smo-related constructs and immunolabeled with cleaved caspase-3 and NeuN antibodies. Nuclei are stained with DAPI. Scale bar: 100 µm. (D) Ratio of cleaved caspase-3-positive neurons (apoptotic) per surface area and expressed as a percentage of NeuN-positive cells. Overexpression of Smo-CA or Smo-ΔN does not increase the number of apoptotic neurons compared to control (GFP). Errors bars are mean±s.d. Number of slices and rats are indicated in parenthesis. *P<0.05; ****P<0.0001 (Mann–Whitney test).

In hippocampal neurons, exogenous treatment with Shh or Smo agonist (SAG) upregulates presynaptic nerve terminals (Mitchell et al., 2012). These observations led us to investigate whether Smo-CA or Smo-ΔN could modulate the density of presynaptic terminals in electroporated cortices. To assess the possible changes at the presynaptic level, we performed quantitative analysis of the area fraction of synaptophysin on MAP2-positive neurons at postnatal day 30 (P30). Compared with control tissues, Smo-ΔN-electroporated tissues showed a significant decrease in the area fraction of synaptophysin immunolabeling (median of 16.05% for Smo-ΔN versus 19.67% for control; *P*=0.01, Mann–Whitney test; Fig. 2A,B), whereas Smo-CA electroporation increased the area fraction of synaptophysin-positive terminals (24.77% for Smo-CA; *P*=0.01 when compared to control and *P*<0.0001 when compared to Smo-ΔN, Mann–Whitney test; Fig. 2B).

Recent evidence indicates that Smo signaling inhibits neuronal apoptosis (Wang et al., 2018), whereas loss of Smo activity leads to increased neuronal apoptosis (Qin et al., 2019). In order to assess whether Smo-CA or Smo-ΔN induces apoptosis in electroporated neurons, we performed immunohistochemical analysis to detect cleaved caspase-3, a marker for cell apoptosis (Logue and Martin, 2008) on electroporated rats at P15. Quantification of cleaved caspase-3-positive neurons showed no significant difference between the constitutively active (mean±s.d. of 0.24±0.13% and 0.34±0.23% in Smo-CA and control, respectively; *P*=0.70, Mann–Whitney test; Fig. 2C,D) or negative-phenotype (0.23±0.15; *P*=0.74, Mann–Whitney test; Fig. 2C,D) forms of Smo when compared to control tissues.

These results suggest that IUE of Smo-related constructs does not affect apoptosis mechanisms in the postnatal cerebral cortex. By contrast, Smo-CA and Smo-ΔN significantly change the formation of synaptic terminals. These observations are in accordance with previous findings showing Smo-dependent enhancement of synapse formation in hippocampal neurons (Mitchell et al., 2012).

### Smo-related constructs regulate the expression of downstream Smo target genes

**Fig. 3.**
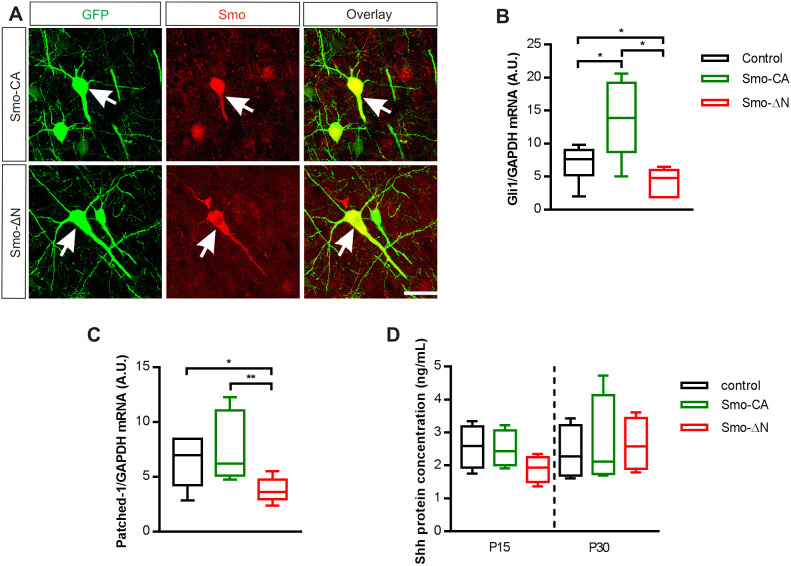
**Smo-related constructs regulate the expression levels of Ptch1 and Gli1.** (A) Cells overexpressing Smo-related constructs (green) and immunostained with Smo antibody (red) showed a similar localization (overlay). Arrows indicate co-localization of Smo and GFP. Scale bar: 25 µm. (B,C) Box plots of relative expression of *Gli1* and *Ptch1* mRNA transcripts in GFP- (control), Smo-CA- and Smo-ΔN-expressing rats. Expression is normalized to GAPDH expression in the same RNA preparation. *n*=6 rats for each condition. (D) Shh is expressed in post-natal somatosensory cortex. Box plots show median Shh protein concentration measured by ELISA at P15 and P30 in somatosensory cortex tissue lysate from GFP- (control), Smo-CA- and Smo-ΔN-expressing rats. *n*=4 rats for each condition. Box plots show the interquartile range with the median indicated. Whiskers show the range. *P<0.05; **P<0.01 (Mann–Whitney test).

To investigate the functional expression of Smo mutants, we performed immunofluorescence labeling in somatosensory cortical tissues overexpressing Smo-related constructs at P15 using an antibody raised against Smo protein. As illustrated in Fig. 3A, the anti-Smo antibody detected the expression of Smo-CA and Smo-ΔN in the electroporated somatosensory cortex. Smo activation mobilizes activator forms of Gli transcription factors, which in turn lead to an increase in Ptch1 and Gli1 expression (Jacob and Lum, 2007). To validate functional expression of the Smo-related constructs, we quantified *Gli1* and *Ptch1* mRNA in cortices overexpressing Smo-CA or Smo-ΔN mutants using reverse transcriptase quantitative PCR (RT-qPCR). As expected, *Gli1* mRNA expression was increased in Smo-CA-expressing animals when compared with levels in GFP-expressing control animals [median of 1.39 arbitrary units (a.u.) versus 0.76 a.u. in Smo-CA and control, respectively; *P*=0.041, Mann–Whitney test; Fig. 3B] and, conversely, was decreased in Smo-ΔN-expressing animals (0.48 a.u.; *P*=0.039 when compared to control, Mann–Whitney test; Fig. 3B). When compared with levels in control or Smo-CA-expressing rats, *Ptch1* mRNA transcripts were downregulated in Smo-ΔN-expressing rats (3.61 a.u. in Smo-ΔN versus 6.97 a.u. in control and 6.21 a.u. in Smo-CA; *P*=0.026 and *P*=0.008, respectively; Mann–Whitney test; Fig. 3C). No significant difference was observed between *Ptch1* mRNA levels in control and Smo-CA-expressing rats (6.21 a.u. for Smo-CA; *P*=0.66, Mann–Whitney test; Fig. 3C). We next measured Shh protein expression levels in the somatosensory cortex of GFP-, Smo-CA- and Smo-ΔN-expressing animals at P15 and P30 by ELISA. Our results indicated that Shh protein was expressed at a constant and similar level in all samples analyzed (2.58 ng/ml for control versus 2.42 ng/ml for Smo-CA and 1.93 ng/ml for Smo-ΔN at P15, *P*>0.05; 2.27 ng/ml for control versus 2.11 ng/ml for Smo-CA and 2.57 ng/ml for Smo-ΔN at P30, *P*>0.05; Mann–Whitney test; Fig. 3D).

We show here that, in electroporated somatosensory cortices, the constitutively active form of Smo significantly promotes the expression of the target gene *Gli1*, whereas the negative-phenotype form of Smo acts in the opposite way by reducing *Gli1* expression.

### Smo controls the developmental GABA excitatory-to-inhibitory shift

**Fig. 4.**
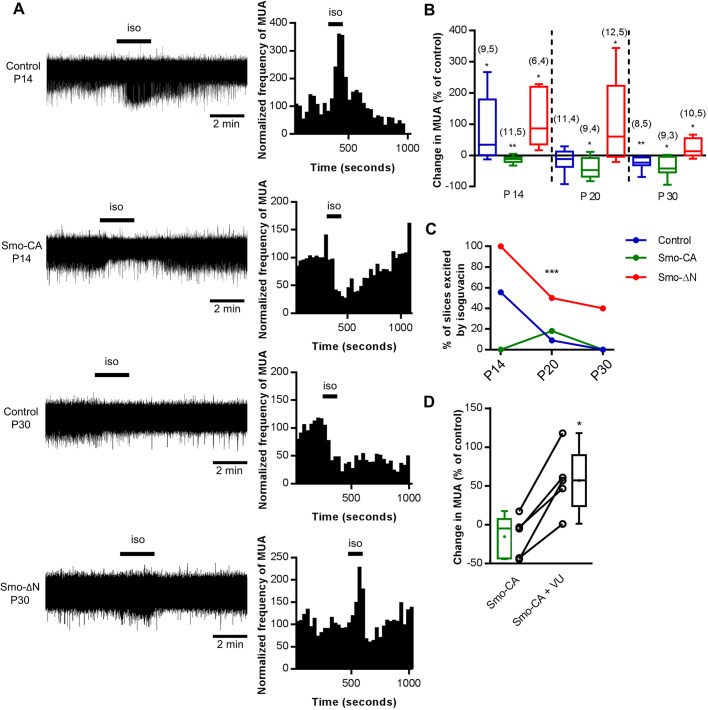
**The developmental shift in the polarity of GABA_A_R-mediated responses is modulated in cortical neurons expressing the negative-phenotype or the constitutively active forms of Smo.** (A) Effects of isoguvacine treatment (iso; 10 µM) in cortical slices from control GFP-, Smo-CA- and Smo-ΔN-expressing rats. Representative traces of spontaneous extracellular field potentials (left column) recorded in the electroporated area of cortical slices at P14 in control and Smo-CA-expressing rats and at P30 in control and Smo-ΔN-expressing rats. Corresponding time course of normalized frequency of MUA is shown (right column). (B) Box plots of relative change of isoguvacine-dependent MUA frequency in electroporated rats at P14, P20 and P30. Numbers in parenthesis indicate the number of slices recorded and rats used. *P<0.05, **P<0.01 compared to control baseline; Wilcoxon test. (C) Developmental regulation of GABAergic excitation in electroporated rats at P14, P20 and P30. Proportion of excited slices is the proportion of slices showing an increase in MUA frequency of 20% or more during isoguvacine application. 3–5 rats per age and condition. ***P<0.001; Chi-squared test. (D) Box plots of relative changes in MUA frequency during isoguvacine application with and without application of VU0463271 (VU), a KCC2-selective blocker, on Smo-CA-expressing rats at P14. *n*=5 rats. Box plots show the interquartile range with the median indicated. Whiskers show the range. *P<0.05 (Wilcoxon test).

Cortical neuronal network construction during the postnatal period requires the depolarizing-to-hyperpolarizing shift of GABA to occur with precise timing during maturation (Ben-Ari and Holmes, 2005; Wang and Kriegstein, 2009; Wu and Sun, 2015). Because Shh signaling plays a nodal role in brain development (Álvarez-Buylla and Ihrie, 2014; Delmotte et al., 2020; Yao et al., 2016), we investigated whether the manipulation of Smo activity affects the onset of the GABA excitatory-to-inhibitory postnatal shift at the network level. We performed field recordings of multiunit activity (MUA) in acute cortical slices from control (GFP), Smo-CA- and Smo-ΔN-expressing rats and measured the effect of bath application of the GABA_A_ receptor (GABA_A_R) agonist isoguvacine (10 µM) on their spiking activity at P14, P20 and P30. In accordance with the known depolarizing action of GABA in the immature neocortex (Kirmse et al., 2015; Riffault et al., 2018), isoguvacine induced an increase of spiking activity at P14 in cortical slices from control and Smo-ΔN-expressing rodents [median of +33.87% (*P=*0.019) and +86.12% (*P=*0.019), respectively; Wilcoxon matched-pairs signed test; Fig. 4A,B]. In contrast, isoguvacine decreased the spiking activity in Smo-CA-expressing animals (−12%; *P=*0.009, Wilcoxon matched-pairs signed test; Fig. 4A,B). At P20, isoguvacine induced either a decrease or an increase of spiking activity in control (−11.37%; *P=*0.519, Wilcoxon matched-pairs signed test; Fig. 4B). In slices obtained from Smo-CA-expressing rats we observed a decrease in spike frequency (−47.03%; *P*=0.011, Wilcoxon matched-pairs signed test), whereas in Smo-ΔN-expressing rats, isoguvacine treatment increased the spike frequency (+60.21%; *P*=0.042, Wilcoxon matched-pairs signed test). At P30 (Fig. 4A–C), isoguvacine induced a decrease in spike frequency in control and Smo-CA-expressing cortical slices [−22.86% (*P=*0.008) and −42.15% (*P*=0.015), respectively; Wilcoxon matched-pairs signed test], whereas an increase in spiking activity was observed in Smo-ΔN-expressing rats (+12.89%; *P*=0.037, Wilcoxon matched-pairs signed test). Thus, the overall effects of isoguvacine treatment on spiking activity suggest that the constitutively active and the negative-phenotype forms of Smo respectively accelerate and delay the developmental hyperpolarizing GABA shift (*P*<0.0001, Chi-squared test; Fig. 4C). In developing neocortex, the depolarizing-to-hyperpolarizing shift of GABA depends primarily on enhancement of the functional expression of KCC2 (Ben-Ari et al., 1989; Rivera et al., 1999). To investigate whether the hyperpolarizing shift of GABA observed in Smo-CA-expressing rats might involve activation of KCC2 we applied the KCC2-selective blocker VU0463271 (VU) on cortical slices obtained from Smo-CA-expressing rats at P14. VU application shifted the isoguvacine response from inhibitory to excitatory when compared with the response of non-treated Smo-CA-expressing slices (−12% without VU versus +57.1% with VU; *P*=0.0009, Mann–Whitney test; Fig. 4D).

Collectively, our data suggest that increasing Smo activity prematurely shifts GABA polarity from excitatory to inhibitory in a KCC2-dependent manner, whereas inhibiting Smo signaling delays the switch in GABA polarity.

### Smo signaling controls chloride homeostasis

**Fig. 5.**
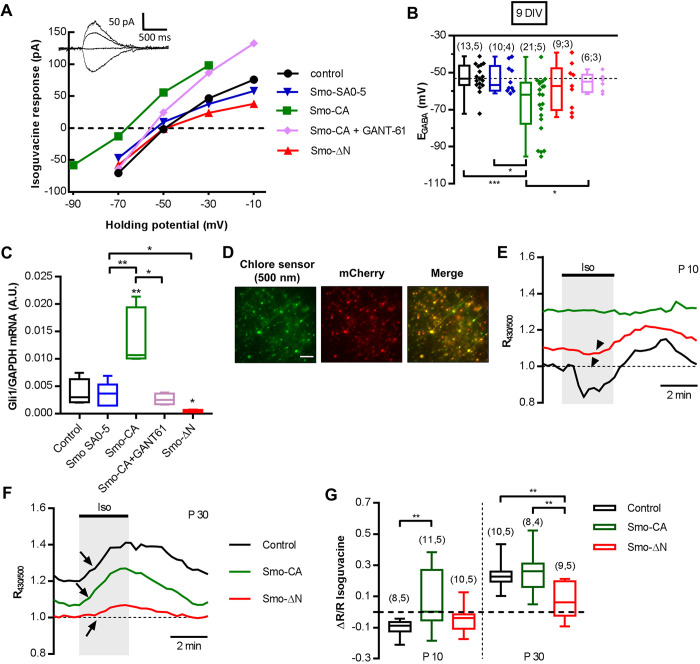
**Smo signaling controls chloride homeostasis in cultured neurons and acute slices.** (A) Representative gramicidin-perforated patch-clamp recordings of current–voltage (I–V) relationships for isoguvacine currents in rat hippocampal primary neuronal cultures (9 DIV) expressing mCherry only (control) or mCherry plus Smo-SA0-5, mCherry plus Smo-CA (with or without GANT-61 preincubation) or Smo-ΔN. Inserts depict the isoguvacine currents for the control condition. (B) Box plots of E_GABA_ in the indicated conditions, color-coded as in A. Individual points are shown alongside the box plots. Dashed line shows the median value of E_GABA_ for the control condition. The number of cells recorded and cultures used are indicated in parenthesis. (C) Box plots of *Gli1* mRNA level in the indicated transfected primary neurons, measured by single-cell RT-qPCR (15 cells per culture per condition, *n*=4 cultures). (D) Typical images of Cl-Sensor fluorescence excited at 500 nm in a slice from a P30 rat electroporated with Cl-Sensor plus Smo-CA. Rats were electroporated *in utero* at E15 with Cl-Sensor plus mCherry (control), Cl-Sensor plus Smo-CA (Smo-CA) or Cl-Sensor plus Smo-ΔN (Smo-ΔN). For analysis, regions of interest were drawn around the soma of cells located in the focal plane. Five to ten neurons were analyzed per image. Scale bar: 100 μm. (E,F) Example of typical ratiometric fluorescence (R_430/500_) recordings at P10 (E) and P30 (F) from control, Smo-CA and Smo-ΔN rats. Horizontal bars indicate the times of application of ACSF containing isoguvacine (iso). Arrowheads and arrows indicate different types of responses between control, Smo-CA and Smo-ΔN neurons at P10 and P30, respectively. Data represent results obtained from four or five rats per experimental condition and one or two slices were recorded per animal. (G) Box plots showing quantification of fluorescence change of ΔR/R, where R is the mean of five R_430/500_ measurements before isoguvacine application and ΔR is the difference between R and the absolute maximum of isoguvacine-induced response. Number of slices and rats are indicated in parenthesis. Box plots in B, C and G show the interquartile range with the median indicated. Whiskers show the range. *P<0.05, **P<0.01, ***P<0.001 (Mann–Whitney test).

To further link Smo signaling with the shift in GABA polarity and neuronal chloride homeostasis, we used the gramicidin-perforated patch-clamp technique to measure GABA_A_ reversal potential (E_GABA_) in dissociated hippocampal cultures of immature neurons [9 days *in vitro* (DIV)]. To ensure that the overexpression of Smo-related constructs did not modify E_GABA_ by itself, we compared the values of E_GABA_ for neurons transfected with Smo SA0-5, a constitutively inactive form of Smo (Chen et al., 2011), and control neurons transfected with mCherry, and found that they were identical (median of −56.68 mV for Smo SA0-5 versus −53.16 mV for mCherry; *P*=0.50, Mann–Whitney test; Fig. 5B). Consistent with our results obtained in somatosensory cortical slices, the measurements of E_GABA_ in Smo-CA-transfected neurons showed more hyperpolarized values when compared to control mCherry-transfected neurons and Smo-SA0-5-transfected neurons [−61.9 mV in Smo-CA versus −53.16 mV in control (*P*=0.0004) and −56.68 mV in Smo SA0-5 (*P*=0.02); Mann–Whitney test; Fig. 5A,B]. Contrary to action of Smo-CA, overexpression of Smo-ΔN did not produce a statistically significant change of E_GABA_, as compared to that of control mCherry-transfected neurons (–57.13 mV in Smo-ΔN; *P*=0.45, Mann–Whitney test; Fig. 5B). To investigate further whether Smo acts through the canonical Gli-dependent pathway, we used the Gli antagonist GANT61, a downstream inhibitor of the canonical Shh–Smo signaling pathway (Lauth et al., 2007). We found that application of GANT61 abolished the hyperpolarized E_GABA_ values observed in Smo-CA-expressing neurons, restoring it to values similar to those observed in control neurons (−54.92 mV in Smo-CA with GANT61 versus −61.9 mV for Smo-CA and −53.16 mV for the control; *P*=0.04 and *P*=0.36, respectively; Mann–Whitney test; Fig. 5A,B).

Next, we used single cell RT-qPCR to quantify *Gli1* mRNA levels in Smo-transfected neurons, with or without the application of 10 µM GANT61. In Smo-CA-transfected cells, *Gli1* mRNA levels were significantly increased compared to levels in control and Smo SA0-5-transfected cells (0.01 a.u. in Smo-CA versus 0.0029 a.u. in control and 0.0036 a.u. in Smo SA0-5; *P*=0.008 and *P*=0.008, respectively; Mann–Whitney test; Fig. 5C). Conversely, *Gli1* mRNA levels were downregulated in Smo-ΔN-transfected neurons (0.0004 a.u. in Smo-ΔN versus 0.0029 a.u. in control and 0.0036 a.u. in Smo SA0-5; *P*=0.015 and *P*=0.016, respectively; Mann–Whitney test; Fig. 5C). Moreover, the effect of Smo-CA on the target gene *Gli1* was blocked in the presence of GANT61 (0.0024 a.u. in Smo CA with GANT61; *P*=0.016 when compared to Smo-CA alone; *P*=0.41 and *P*>0.99 when compared with control and Smo SA0-5, respectively; Mann–Whitney test; Fig. 5C). These results, therefore, suggest that the constitutively active form of Smo prematurely shifts GABA polarity through the activation of the canonical Gli-dependent transcription pathway.

To corroborate the results obtained in neuronal cultures, we studied whether Smo signaling modifies intracellular chloride concentration ([Cl^−^]_i_) in acute slices of somatosensory cortex isolated from Smo-CA- and Smo-ΔN-expressing rats. For this purpose, mCherry (control), Smo-CA or Smo-ΔN were co-electroporated *in utero* at E15 with constructs encoding a ratiometric chloride sensor (Cl-Sensor; Friedel et al., 2013, 2015) (Fig. 5D). Electroporated slices prepared at P10 and P30 harbored hundreds of neurons expressing Cl-Sensor in cortical layers V and VI. Bath application of isoguvacine (10 µM, 3 min) to P10 Smo-ΔN-expressing and control slices produced a uniform decrease of the fluorescence intensity ratio R_430/500_ (indicated by an arrowhead in Fig. 5E and by the negative value of ΔR/R in Fig. 5G), reflecting a chloride ion extrusion characteristic of depolarizing action of GABA (median of −0.04 a.u. for Smo-ΔN versus −0.09 a.u. for control; *P*=0.17, Mann–Whitney test; Fig. 5E,G), whereas in Smo-CA-expressing slices, isoguvacine triggered either an increase, no change or slight decrease of R_430/500_. The median of these changes in Smo-CA slices was 0.00 a.u., and statistical analysis revealed significant difference when compared to control (*P*=0.005, Mann–Whitney test; Fig. 5E,G). This difference indicates that in Smo-CA-expressing slices GABA is switching from depolarizing to hyperpolarizing actions. In control and Smo-CA-expressing slices at P30, isoguvacine produced a chloride influx (indicated by an arrow in Fig. 5F) as shown by the positive value of ΔR/R (+0.22 a.u. for control versus +0.26 a.u. for Smo-CA; *P*=0.63, Mann–Whitney test; Fig. 5F,G), whereas Smo-ΔN-expressing slices showed a slight increase of R_430/500_ following isoguvacine treatment (+0.06 a.u.; *P*=0.002 and *P*=0.01 when compared to control and Smo-CA, respectively; Fig. 5F,G). The latter finding suggests that a Smo-dependent pathway is active at rest in mature P30 neurons to maintain the low [Cl^−^]_i_ and hyperpolarizing inhibitory action of GABA. The inhibition of Smo action using Smo-ΔN led to an increase in [Cl^−^]_i_ that reduces the inhibitory strength of GABA.

Thus, in both immature acute slices and primary cultures, activation of the Smo signaling pathway using Smo-CA led to a hyperpolarizing shift of Cl^−^, whereas Smo-ΔN did not affect the [Cl^−^]_i_ at this stage.

### Smo regulates the activity of KCC2

**Fig. 6.**
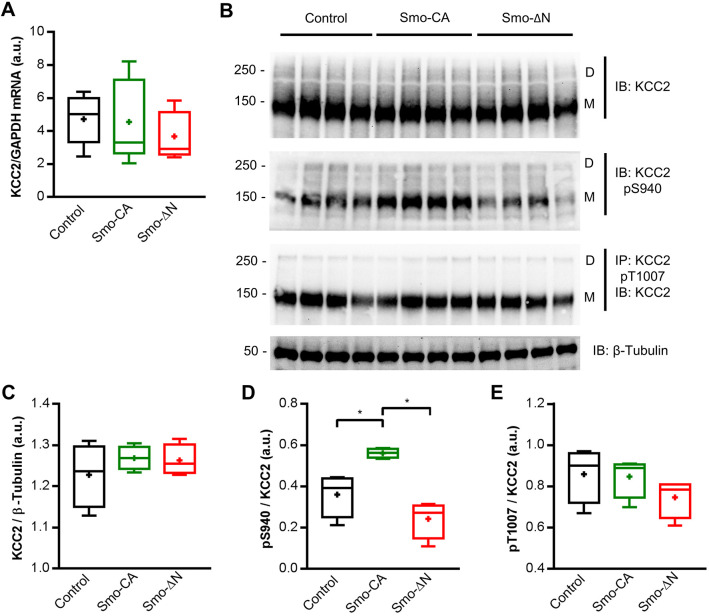
**KCC2 Ser^940^ phosphorylation is regulated by Smo signaling.** (A) Box plots of relative expression of KCC2 mRNA normalized to GAPDH mRNA level in P10 rats electroporated with GFP alone (control), GFP and Smo-CA (Smo-CA) or GFP and Smo-ΔN (Smo-ΔN). *n*=5 rats for each condition. (B) Immunoblots for total KCC2 and phosphorylated forms of KCC2 Ser^940^ (KCC2 pS940) and KCC2 Thy^1007^ (KCC2 pT1007) in protein extracts from P10 electroporated somatosensory cortices. Molecular weights are indicated on the left of the blot (in kDa). β-tubulin is shown as a loading control. D, dimer; M, monomer; IB, immunoblot; IP, immunoprecipitation. (C–E) Box plots of normalized total KCC2(C), KCC2 pSer^940^ (D) and KCC2 pThr^1007^ (E) protein level. *n*=4 rats for each condition. Box plots show the interquartile range with the median indicated by a line and the mean indicated by a plus symbol. Whiskers show the range. *P<0.05 (Mann–Whitney test).

Regulation of the KCC2-dependent developmental shift of GABA relies on a complex mechanism involving a progressive increase in the amount of KCC2 protein and its posttranslational modifications via at least two distinct phosphorylation sites, serine 940 (Ser^940^) and threonine 1007 (Thr^1007^) (Côme et al., 2019; Medina et al., 2014; Moore et al., 2017). We measured the protein and mRNA expression of KCC2 from the somatosensory cortices of Smo-CA- and Smo-ΔN-expressing rats at P10. We observed similar levels in control rats and rats expressing the Smo-related constructs for KCC2 mRNA (median of 5.02 a.u. for control tissues versus 3.3 a.u. for Smo-CA and 2.92 a.u. for Smo-ΔN; *P*=0.84 and *P*=0.42, respectively, compared to control; Mann–Whitney test; Fig. 6A) and protein (1.23 a.u. for control tissues when normalized to β3 tubulin versus 1.26 a.u. for Smo-CA and 1.25 a.u. for Smo-ΔN; *P=*0.48 and *P=*0.48, respectively; Mann–Whitney test; Fig. 6B,C). The membrane stability and transporter activity of KCC2 are dependent on the phosphorylation state of intracellular C-terminal domains. For instance, phosphorylation of the Ser^940^ residue increases stability and functionality of KCC2, whereas phosphorylation on the Thr^1007^ residue enhances KCC2 endocytosis (Lee et al., 2007; Medina et al., 2014). To determine which steps in KCC2 trafficking may be regulated by Smo-related constructs, we carried out a quantitative immunoblotting assay using antibodies recognizing the Ser^940^ (Lee et al., 2011) and Thr^1007^ (de Los Heros et al., 2014) phosphorylated forms of KCC2. We found that the ratio of phospho-Ser^940^ (pKCC2 S^940^) to total KCC2 protein (pKCC2 S^940^/KCC2) was upregulated in Smo-CA-electroporated cortices when compared to the ratio in control and Smo-ΔN conditions (0.56 a.u. for Smo-CA versus 0.39 a.u. for control and 0.27 a.u. for Smo-ΔN; *P*=0.02 and *P*=0.02, respectively, when compared to Smo-CA; Mann–Whitney test; Fig. 6B,D). The ratio of phospho-Thr^1007^ (pKCC2 Thr^1007^) to total KCC2 protein (pKCC2 T^1007^/KCC2) was not modified in cortices electroporated with Smo-related constructs (0.90 a.u. for control tissues versus 0.89 a.u. for Smo-CA and 0.78 a.u. for Smo-ΔN; *P*=0.88 and *P*=0.17, respectively, when compared to control; Mann–Whitney test; Fig. 6B,E).

Taken together, these data suggest that enhanced Smo activity led to KCC2 posttranslational modifications, with increased Ser^940^ phosphorylation in Smo-CA-electroporated somatosensory cortex.

### The negative-phenotype form of Smo affects the stability of KCC2-pH_ext_ at the plasma membrane by promoting endocytosis

The phosphorylation of Ser^940^ described above is well known for its ability to stabilize KCC2 in the plasma membrane, whereas dephosphorylation of Ser^940^ promotes KCC2 endocytosis (Kahle et al., 2013; Lee et al., 2007). We therefore assessed whether Smo-related constructs are able to regulate KCC2 trafficking to the cell surface. As a tool, we used a KCC2 construct tagged in an external loop with the fluorescent protein pHluorin (KCC2-pH_ext_) (Kahle et al., 2014). This construct allows both measurement of the KCC2-pH_ext_-dependent shift of [Cl^−^]_i_ and visualization of surface expression and/or internalization of KCC2-pH_ext._ (Friedel et al., 2015, 2017).

**Fig. 7.**
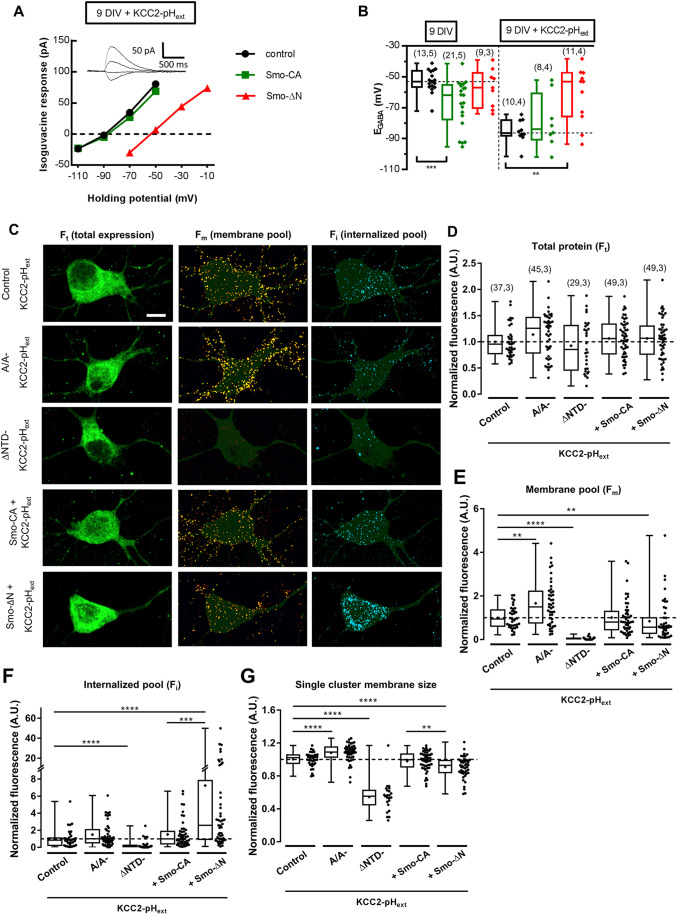
**The negative-phenotype form of Smo affects the stability of KCC2 at the plasma membrane surface.** (A) Gramicidin-perforated patch-clamp recording current–voltage (I–V) relationships for isoguvacine-response currents in hippocampal primary cultures at 9 DIV transfected with KCC2-pH_ext_ alone (control, black) or co-transfected with Smo-CA (green) or Smo-ΔN (red). Inserts depict the isoguvacine currents for the control condition. (B) Box plots of E_GABA_ in the indicated conditions, color-coded as in A. Left panel shows neurons transfected without KCC2-pH_ext_ from data shown in Fig. 4A,B. Right panel shows neurons co-transfected with KCC2-pH_ext_. The number of cells recorded and cultures used are indicated in parenthesis. Dashed line shows the median value for the control condition. (C) Representative images illustrating total, membrane and internalized pools of KCC2 with an external tag (KCC2-pHext) in vehicle (control), and Smo-related transfected constructs in hippocampal primary culture neurons. A KCC2 mutant with stable membrane surface expression (A/A-KCC2-pH_ext_) was used as positive control for KCC2 membrane trafficking. Neurons expressing a KCC2 mutant known to not be trafficked to the membrane (ΔNTD-KCC2-pH_ext_) were used in parallel experiments to ensure that immunocytochemistry on living neurons did not permeabilize the membrane. Scale bar: 20 μm. (D) Box plots of total protein (F_t_), (E) normalized membrane (F_m_) and (F) internalized pool (F_i_) fluorescence, and of (G) single membrane cluster size in cultured neurons expressing the indicated constructs. The number of cells and cultures used in D–G are indicated in parentheses in D and are identical for all box plots. Box plots show interquartile range with median indicated. The whiskers show the range. Individual data points are shown alongside the box plots. *P<0.05, **P<0.01, ***P<0.005, ****P<0.001 (Mann–Whitney test).

As expected, overexpression of KCC2-pH_ext_ in neurons at 9 DIV resulted in a negative shift of E_GABA_ (median of −53.16 mV in control neurons and −86.25 mV in neurons overexpressing KCC2-pH_ext_; *P*<0.001, Mann–Whitney test; Fig. 7A,B), indicating also that at least some portion of KCC2-pH_ext_ molecules were delivered to the plasma membrane. The overexpression of Smo-CA did not produce additional hyperpolarizing shift of E_GABA_ in KCC2-pH_ext_-positive neurons (-83.82 mV for Smo-CA and KCC2-pH_ext_ versus −86.25 mV for mCherry and KCC2-pH_ext_; *P*=0.69, Mann–Whitney test; Fig. 7A,B), whereas overexpression of Smo-ΔN resulted in a significant depolarizing shift of this parameter (−53.1 mV in Smo-ΔN and KCC2-pH_ext_ versus −86.25 mV in mCherry and KCC2-pH_ext_; *P*=0.006, Mann–Whitney test; Fig. 7A,B) indicating either a Smo-ΔN-dependent inactivation of the membrane pools of KCC2-pH_ext_ or reduction of KCC2-pH_ext_ expression at the plasma membrane.

To corroborate these observations, we performed a live-staining analysis of surface-expressed and internalized KCC2-pH_ext_. Three different pools of KCC2-pH_ext_ were revealed using a multistep immunolabeling protocol: the total expressed amount of protein (F_t_), the amount of KCC2-pH_ext_ cell surface expression (F_m_) and the amount of internalized KCC2-pH_ext_ (F_i_) (Fig. 7C). As positive and negative controls we used overexpression of KCC2-pH_ext_ mutants T906A/T1007A (A/A-KCC2-pH_ext_) and ΔNTD-KCC2-pH_ext_, which are known for their increased and perturbed surface expression abilities, respectively (Friedel et al., 2017). Live-cell immunolabeling of the control non-mutated KCC2-pH_ext_ revealed a well detectable F_m_ signal in the form of clusters, whereas the post-hoc multistep immunolabelings revealed the F_i_ pool of molecules and the total amount of expressed KCC2-pH_ext_ (Fig. 7C). The five tested constructs showed similar intensities of F_t_ (median of 0.95 a.u. for KCC2-pH_ext_, 1.25 a.u. for A/A-KCC2-pH_ext_, 0.85 a.u. for ΔNTD-KCC2-pH_ext_, 1.062 a.u. for Smo-CA with KCC2-pH_ext_, and 1.067 a.u. for Smo-ΔN with KCC2-pH_ext_; Fig. 7D). The relative amounts of F_m_ and the membrane-labeled cluster size in KCC2-pH_ext_-expressing neurons were lower than those in A/A-KCC2-pH_ext_-expressing neurons and significantly higher than the values revealed in ΔNTD-KCC2-pH_ext_-transfected cells (for F_m_: 0.94 a.u. for KCC2-pH_ext_ versus 1.49 a.u. for A/A-KCC2-pH_ext_ and 0.008 a.u. for ΔNTD-KCC2-pH_ext_; *P*=0.002 and *P*<0.0001, respectively; Fig. 7E; for single cluster membrane size: 1.02 a.u. for KCC2-pH_ext_ versus 1.09 a.u. for A/A-KCC2-pH_ext_ and 0.55 a.u. for ΔNTD-KCC2-pH_ext_; *P*<0.0001 and *P*<0.0001, respectively; Mann–Whitney test; Fig. 7G). Co-expression of Smo-CA with KCC2-pH_ext_ did not produce a statistically significant change in F_i_, F_m_ or single surface-labeled cluster size (Fig. 7E–G). By contrast, neurons co-expressing KCC2-pH_ext_ and Smo-ΔN showed a significant twofold higher internalization rate of labeled KCC2-pH_ext_ molecules (0.84 a.u. for KCC2-pH_ext_ versus 2.51 a.u. for Smo-ΔN with KCC2-pH_ext_; *P*<0.0001; Mann–Whitney test; Fig. 7C,F), a significant twofold lower amount of F_m_ (0.94 a.u. for KCC2-pH_ext_ versus 0.57 a.u. for Smo-ΔN with KCC2-pH_ext_; *P*=0.009; Mann–Whitney test; Fig. 7C,E) and significantly smaller size of surface-located KCC2-pH_ext_ clusters (1.02 a.u. for KCC2-pH_ext_ versus 0.92 a.u. for Smo-ΔN with KCC2-pH_ext_; *P*<0.0001, Mann–Whitney test; Fig. 7G).

Thus, consistent with data illustrated in Figs 4–7, both [Cl^−^]_i_ measurement and live-cell immunolabeling revealed a striking Smo-dependent change in function and surface expression of KCC2-pH_ext_.

### Smo-ΔN-expressing rats display increased susceptibility to pentylenetetrazol-induced seizures

**Fig. 8.**
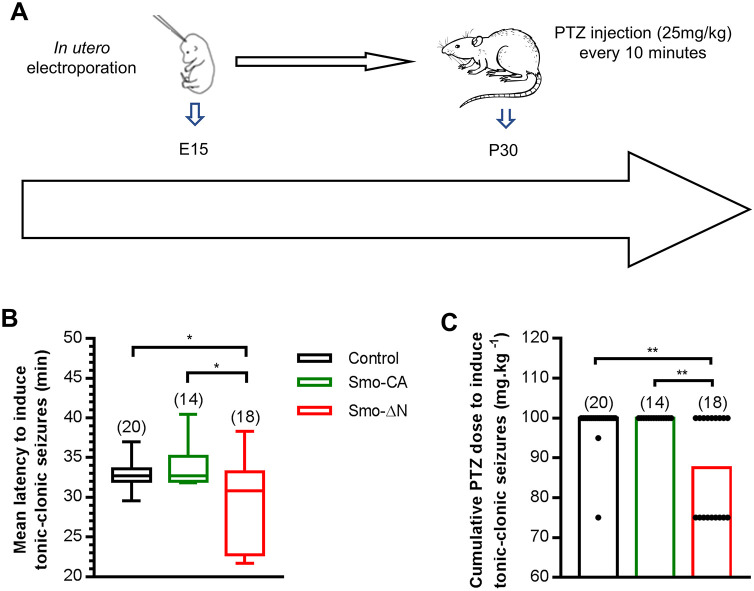
**Modified susceptibility to pentylenetrazol-induced seizures in Smo mutant rats.** (A) Experimental paradigm design. (B) Box plots of latency needed to induce generalized tonic–clonic seizures in three groups of electroporated rats: control (electroporated with GFP), Smo-CA-expressing and Smo-ΔN-expressing. Box plots show interquartile range with median indicated. The whiskers show the range. (C) Bar graph of PTZ cumulative dose needed to induce generalized tonic–clonic seizures. Bars are medians with experimental points shown. Numbers in parenthesis indicate the number of rats used. *P<0.05; **P<0.01 (Mann–Whitney test).

Large number of studies have illustrated that KCC2 dysfunction facilitates initiation of epileptic seizures (Chen et al., 2017; Moore et al., 2018). Changes in KCC2 Ser^940^ phosphorylation that modify neuronal chloride homeostasis and depolarizing strength of GABA strongly affect seizure susceptibility in KCC2 transgenic mice (Silayeva et al., 2015). Finally, we investigated whether overexpression of Smo mutants may influence the susceptibility to seizures. Rats expressing Smo-ΔN or Smo-CA and age-matched control rats (transfected with GFP alone) at P30 were intraperitoneally injected with subconvulsive doses of pentylenetetrazol (PTZ; 25 mg/kg; Fig. 8A), an inhibitor of the GABA_A_ receptors (Klioueva et al., 2001). Previous studies have shown that the lowest threshold susceptibility to PTZ can be observed during the first 2 postnatal weeks, and the highest at around P30 (Klioueva et al., 2001; Vernadakis and Woodbury, 1969). These results suggest that the vulnerability to PTZ is correlated to the maturation of the GABAergic system during postnatal development. Injections of PTZ were continued every 10 min until each rat had generated a generalized tonic–clonic seizure. Seizure susceptibility and severity were quantified using the canonical Racine's scale of seizure severity (Lüttjohann et al., 2009), including dose and latency to onset of the first generalized tonic–clonic seizure. Generalized seizures in Smo-ΔN-expressing rats occurred at lower doses of PTZ (median of 87.5 mg/kg compared with 100 mg/kg for both control and Smo-CA-expressing rats; *P*=0.005 and *P*=0.003, respectively; Mann–Whitney test; Fig. 8C) and with shorter latencies than in control and Smo-CA-expressing rats (30.83 min compared with 32.71 min in control and 32.69 min in Smo-CA; *P*=0.041 and *P*=0.024, respectively; Mann–Whitney test; Fig. 8B). No significant difference was observed between control and Smo-CA-expressing rats in the latency (32.69 min compared with 32.71 min in control; *P=*0.5158; Fig. 8B) or cumulative dose of PTZ to induce tonic–clonic seizures (*P*=0.501; Fig. 8C).

These results indicate that the reduced seizure threshold in Smo-ΔN-expressing rats could be due to a downregulation of KCC2 and deregulation of chloride homeostasis induced by the reduction of Smo activity.

## DISCUSSION

In this study, we investigated the actions of the Smo signaling pathway on GABAergic transmission *in vivo* and *ex vivo* in the rat somatosensory cortex and *in vitro* in primary cultures of hippocampal neurons using negative-phenotype (Smo-ΔN) and constitutively active (Smo-CA) forms of Smo. We show that activation of Smo signaling in immature neurons at early postnatal stages accelerated the GABAergic switch from depolarizing to hyperpolarizing activity in a KCC2-dependent manner, leading to a low intracellular chloride concentration. Conversely, Smo signaling blockade altered the phosphorylation state and membrane stability of KCC2, weakening the GABAergic inhibition in later stages of development and resulting in an increase of seizure susceptibility. These findings reveal an unexpected function of the G-protein-coupled receptor smoothened on chloride homeostasis regulation and, in particular, on the timing of the GABA shift from depolarizing to hyperpolarizing in the postnatal developing brain.

The postnatal developmental switch in GABA polarity is tightly regulated by the actions of several peptides, including neurotrophic factors (Aguado et al., 2003; Kelsch et al., 2001; Ludwig et al., 2011; Riffault et al., 2018), hypothalamic neurohormones (Leonzino et al., 2016; Spoljaric et al., 2017; Tyzio et al., 2006), central and peripheral hormones (Dumon et al., 2018; Sawano et al., 2013). Hence, the precise timing of the GABAergic polarity shift depends on the balance between factors promoting or delaying the onset of GABAergic inhibitory transmission. It is noteworthy that these factors are developmentally regulated and/or operate within specific time windows to control the functional expression of KCC2 and associated chloride homeostasis and, consequently, the postnatal maturation of GABAergic transmission in the central nervous system. Interestingly, the excitatory action of GABA at early stages of development coincide with the predominance of trophic factors responsible for the inhibition of KCC2 expression and function, such as the immature form of BDNF (proBDNF) (Riffault et al., 2018) and the adipocyte hormone leptin (Dumon et al., 2018). These two factors are expressed shortly after birth and surge during the first postnatal week in rodents (Dumon et al., 2018; Menshanov et al., 2015). They have been shown to promote low KCC2 protein and mRNA expression levels and, consequently, a high intracellular chloride concentration, thus maintaining a depolarizing action of GABA in neonates. The developmental decreases of leptin and proBDNF observed at the end of the first postnatal week in rodents are concomitant with an increase in oxytocin (Leonzino et al., 2016; Tyzio et al., 2006), mature BDNF (mBDNF) (Maisonpierre et al., 1990) and thyroid hormones (Sawano et al., 2013), which in turn upregulate KCC2, leading to a low intracellular chloride concentration and a hyperpolarizing action of GABA. Although we did not observe any difference in the level of Shh proteins between P15 and P30, a previous study demonstrated that Shh expression paralleled the temporal expression of mBDNF in the cortex of rodents, with a nearly undetectable level at birth and a continuous increased expression at both mRNA and protein levels during the two first postnatal weeks (Rivell et al., 2019). This temporal expression of Shh and its signaling partners during postnatal brain development has been shown to play a role in neuronal network construction through axonal growth and synaptic maturation (Yao et al., 2015). Here, we uncover a new critical function of the Shh transducer Smo in regulation of the GABA polarity switch. Indeed, we showed that activation of the GABA_A_ receptor by isoguvacine in immature (P14) Smo-CA-expressing cortical neurons resulted in an influx of chloride and a decrease in spiking activity, whereas mature (P30) negative-phenotype Smo cortical neurons (Smo-ΔN), maintained a depolarizing and excitatory action of GABA. Thus, in the absence of Smo signaling, the GABA shift is delayed, thereby leading to an altered chloride homeostasis and higher susceptibility to seizures, presumably reflecting an unbalanced excitation to inhibition (E/I) ratio. E/I imbalance has been linked to cognitive disorders such as schizophrenia, ASD, depression and epilepsy (Ben-Ari and Holmes, 2005; Brambilla et al., 2003; Kuzirian and Paradis, 2011; Mueller et al., 2015). Abnormal levels of Shh have also been observed in ASD patients, with increased serum level observed in children with ASD, and decreased mRNA level measured in post-mortem adult human brain tissue from autism patients (Choi et al., 2014; Halepoto et al., 2015). In addition, mutations in *PTCHD1*, a gene encoding a protein that displays secondary structures similar to Ptch1, are found in patients with intellectual disability and ASD (Ung et al., 2018). Consistent with a possible role of Shh components in the regulation of the E/I balance, these observations also suggest that an alteration in the Shh–Ptch–Smo pathway might have an effect on the pathophysiological mechanisms involved in ASD. In line with these observations, the role of Shh signaling in neuronal activity has been recently addressed in an elegant study (Hill et al., 2019) showing that the selective disruption of Shh signaling using a conditional knockout of Smo leads to an increase in neuronal excitability of cortical neurons, thus highlighting the importance of Shh–Smo signaling for the regulation of the E/I equilibrium and the construction of functional cortical networks. The results from this study are consistent with our findings showing that Smo signaling blockade increased neuronal network excitability in rodents expressing the negative-phenotype form of Smo. Furthermore, we found that blockade of Smo activity in Smo-ΔN-expressing rodents led to a decrease in KCC2 function and to a reduced threshold for PTZ-induced tonic–clonic seizure. In agreement with a previous study by Chen et al. (2017), these results suggest that downregulation of KCC2 function may contribute to epileptic seizures. The implication of Shh–Smo signaling in epilepsy and seizures has been indirectly addressed in previous studies showing that somatic mutations in Shh pathway genes in humans are associated with hypothalamic hamartoma and drug-resistant epilepsy (Hildebrand et al., 2016; Saitsu et al., 2016). Likewise, Shh expression is increased in hippocampus and neocortex from both human and animal models of temporal lobe epilepsy (TLE) (Fang et al., 2011), thus suggesting a potential relationship between epilepsy and Shh activity. In line with the above studies, Feng et al. (2016) reported that during TLE development, Shh contributes to epileptogenesis through the inhibition of glutamate transporter function leading to abnormal extracellular glutamate levels, whereas the blockade of Shh–Smo signaling reduced the severity of seizures. Taken together, these observations can lead to opposing interpretations regarding the possible interplay between Shh–Smo pathway and epilepsy. These apparent discrepancies could be explained by the treatments utilized (i.e. acute seizure models with PTZ versus spontaneous seizures in the pilocarpine model of chronic TLE), which are able to activate different downstream signaling cascades (Choudhry et al., 2014). Moreover, other studies have found that activation of the Shh–Smo signaling pathway increases the synthesis and secretion of neurotrophic factors like nerve growth factor (NGF) and BDNF (Bond et al., 2013; Chen et al., 2018; Delmotte et al., 2020; Radzikinas et al., 2011), which may have neuroprotective effects in TLE (Bovolenta et al., 2010; Falcicchia et al., 2018; Paradiso et al., 2009). Clearly, additional studies are needed to obtain a deeper knowledge of the cellular and molecular mechanisms linking epilepsy to Shh pathway components.

Importantly, our *in vitro* experiments revealed that in immature neurons of both primary rat hippocampal cultures and rat neocortical acute slices the E_GABA_ shifted towards a more hyperpolarized level in Smo-CA-expressing neurons, indicating a Smo-CA-initiated decrease of neuronal [Cl^−^]_i_. This effect of Smo was blocked by preincubation with the Gli transcription factor inhibitor GANT61, suggesting that in the current study, Smo acts through Gli-dependent canonical signaling (Belgacem et al., 2016) to modulate the reversal potential of GABA. Our previous research demonstrated that Smo also participates in the maturation of GABAergic networks in the postnatal rat hippocampus through a non-canonical pathway (Delmotte et al., 2020). Non-canonical Shh signaling has been shown to acutely modulate Ca^2+^-mediated spontaneous electrical activity in the embryonic spinal cord through a G_αi_ protein (Belgacem and Borodinsky, 2011). In this context, the G_αi_-dependent non-canonical signaling acts as a negative regulator of canonical Shh signaling. The activation of Smo leads to an increase of Ca^2+^ spike frequency, leading to protein kinase A activation and subsequent inhibition of the activator forms of Gli transcription factors (Gli1 and Gli2^A^) concomitant with an enhancement of the repressor forms of Gli (Gli3^R^) in embryonic spinal neurons (Belgacem and Borodinsky, 2015). Here, we show an enhancement of Gli activity upon Smo activation, indicating that the non-canonical Smo signaling pathway is not involved (Belgacem and Borodinsky, 2011, 2015). This highlights the diversity of signaling pathways recruited by the Shh signal transducer Smo among cell types, central nervous structures and developmental stages.

In immature neurons the increased [Cl^−^]_i_ and corresponding depolarizing values of E_GABA_ are determined primarily by effective intrusion of Cl^−^ by sodium-potassium-chloride cotransporter type 1 (NKCC1, also known as SLC12A2) and absence of effective Cl^−^ extrusion by KCC2. The blockage of NKCC1 (Yamada et al., 2004) or activation of KCC2 (Dumon et al., 2018; Inoue et al., 2012; Khirug et al., 2005) both lead to a hyperpolarizing shift of E_GABA_. In this study, we revealed a novel Smo-dependent pathway of KCC2 upregulation during neuronal development. Whether the Smo pathway also regulates NKCC1 activity, as well as other transporters and channels controlling Cl^−^ homeostasis, in immature neurons should be a subject of future studies.

The Cl^−^ extrusion activity of KCC2 in developing neurons depends on protein expression level (Rivera et al., 1999) and posttranslational modifications that regulate the surface targeting and clustering of KCC2 (Côme et al., 2019; Medina et al., 2014). Thus, KCC2 cycles between synaptic sites and endocytic compartments, depending on the phosphorylation state of different amino acid residues (Côme et al., 2019; Kahle and Delpire, 2016; Kahle et al., 2014). However, this membrane turnover also requires regulation by the cytosolic N- and C-termini of KCC2 (Friedel et al., 2017). For instance, N-terminal truncation of KCC2 prevents its export to the cell surface, whereas C-terminal truncation leads to a decreased surface expression of KCC2 (Friedel et al., 2017). The observed loss of KCC2 surface expression in the C-terminal mutants is explained by an increase in the internalization rate without affecting cell-surface delivery. Furthermore, KCC2 function can also be regulated through lateral diffusion at the plasma membrane (Chamma et al., 2013). This suggests that KCC2 membrane dispersion may, in turn, mediate membrane destabilization and endocytosis, resulting in dysregulation of intracellular chloride homeostasis (Côme et al., 2019). In agreement with this, we found that inhibition of the Smo signaling pathway in mature neurons induces a decrease in KCC2 cluster size and enhances the intracellular concentration of chloride, which sets E_GABA_ at more positive values than the resting potential and reduces cell-surface expression levels of KCC2. We further show that these effects occur through an increased rate of KCC2 turnover.

In conclusion, these data uncover an unexpected role for the Shh transducer Smo during early postnatal development through the control of chloride homeostasis. Thus, Smo signaling can tune GABA inhibitory transmission by controlling phosphorylation of KCC2 that affects KCC2 stability in the cell membrane and modulates neuronal chloride homeostasis. Finally, these results suggest that Smo signaling is able to set the cursor of the GABAergic inhibitory switch during the critical maturational period where developmental pathogenesis takes place.

## MATERIALS AND METHODS

Animal procedures

All animal procedures were carried out according to guidelines set by the INSERM animal welfare committee through the local committee (CEEA n°14) and the European Communities Council Directives (2010/63/UE). Male and female Wistar rats were purchased from Janvier Labs (https://www.janvier-labs.com/fiche_produit/rat_wistar/). Animals were raised and mated at INMED A2 animal facility and were housed under a 12 h light/dark cycle at 22–24°C with access to food and water *ad libitum*.

### Reagents and treatments

The following reagents were purchased from the indicated sources: 1,2,3,4-Tetrahydro-6-nitro-2,3-dioxo-benzo[f]quinoxaline-7-sulfonamide (NBQX) was obtained from the Molecular, Cellular and Genomic Neuroscience Research Branch (MCGNRB) of the National Institute of Mental Health (NIMH, Bethesda, MD, USA). Tetrodotoxin (TTX) was purchased from Abcam (Cambridge, UK). Isoguvacine and VU0463271 were purchased from Tocris Cookson (Bristol, UK). Strychnine, GANT61 and bumetanide were from Sigma (St Louis, Missouri, USA).

### *In utero* electroporation

*In utero* injections and electroporations were performed in embryos from timed pregnant rats (embryonic day 15) that received buprenorphine (Buprecare at 0.03 mg/kg) and were anaesthetized with sevoflurane (4.5%) 30 min later. Briefly, the uterine horns were exposed, and a lateral ventricle of each embryo was injected using pulled glass capillaries and a microinjector (PV 820 Pneumatic PicoPump; World Precision Instruments, Sarasota, FL) with Fast Green (2 mg/ml; Sigma, St Louis, MO, USA) combined with the DNA constructs encoding GFP or mCherry and/or Smo-ΔN and/or Smo-CA and/or Cl-Sensor (molar ratio 1:2 between fluorescent protein DNA and other DNA constructs). Cl-sensor constructs was cloned into *gw* vector (Friedel et al., 2015). Smo constructs were provided by Jin Jiang and Philip Beachy (Chen et al., 2011; Kim et al., 2009). Plasmids were further electroporated by delivering 40 V voltage pulses with a BTX ECM 830 electroporator (BTX Harvard Apparatus, Holliston, MA, USA). The voltage was discharged in five electrical pulses at 950-ms intervals via tweezer-type electrodes (Nepa Gene Co, Chiba, Japan) placed on the head of the embryo across the uterine wall. We performed *in utero* electroporation in embryonic rats at E15, corresponding to an active period of both radial and tangential migration of newborn neurons in the cortex (Kriegstein and Noctor, 2004). At birth, successfully electroporated pups were selected after transcranial visualization of the GFP reporter fluorescent protein only. Following experimentation, morphological analysis of electroporated tissues, selected by GFP or mCherry expression under a fluorescence stereomicroscope (Olympus SZX 16), was performed. The criteria commonly used for selection of animals were localization and cell density of transfected cortices and absence of abnormal cortex morphology (i.e. cortical disruption, thickening and abnormal cortical organization). Our analyses revealed no alterations in cortical layer position or morphology in GFP or mCherry, Smo-CA and Smo-ΔN conditions, although we cannot exclude the possibility that Smo constructs impact the neuronal cells at the cellular and subcellular levels. Nevertheless, ∼20% of electroporations failed due to the absence of transfected cells, as revealed by the lack of fluorescent cells (in GFP or mCherry and Smo conditions). These animals were excluded from the study.

### Western blotting

Electroporated zones of the somatosensory cortex were homogenized in RIPA buffer [150 mM NaCl, 1% Triton X-100, 0.1% SDS, 50 mM Tris-HCl, pH 8, containing protease inhibitors (Complete Mini; Roche)]. Lysates were centrifuged (10,000 ***g*** for 10 min at 4°C) and the supernatant was heated at 90°C for 5 min with Laemmli loading buffer. Loading was 20 µg of protein, as determined using a modified Bradford reaction (Bio-Rad Laboratories). Proteins were separated by 7–15% SDS–PAGE and electrophoretically transferred to nitrocellulose membranes. Membranes were blocked with 5% bovine serum albumin (BSA) in TBS containing 0.1% Tween 20 (TBST) for 2 h at room temperature, then incubated with primary antibodies diluted in TBST containing 3% BSA overnight at 4°C or for 2 h at room temperature. Blots were probed with antibodies against KCC2 pSer^940^ (1 mg/ml; rabbit, Novus Biologicals), KCC2 pThr^1007^ (1 mg/ml; sheep, Division of Signal Transduction Therapy Unit, University of Dundee) and KCC2 (1:2000; rabbit, US Biological). After washing with TBST, membranes were incubated with HRP-conjugated secondary antibodies diluted in TBST containing 3% BSA for 60 min, washed with TBST and then developed using a G:BOX gel imaging system (Syngene). The appropriate exposure time of the digital camera for acquisition of chemiluminescent signals from immunoblots was adapted to avoid saturated pixels, and expression levels were estimated using ImageJ software (NIH, Bethesda, MD; http://rsb.info.nih.gov/ij/).

### Immunocytochemistry and confocal microscopy

Under deep anesthesia with isoflurane prior to chloral hydrate (7% in 1 M PBS), P10 to P30 electroporated rats were intracardially perfused with cold PBS (1 M) followed by 4% paraformaldehyde (PFA) in PBS. Brains were removed, post-fixed overnight at 4°C and rinsed in PBS. Coronal cortical sections (70 μm thick) were obtained using a vibratome (Microm HM 650 V). Sections were incubated first for 1 h in PBS with 1% BSA and 0.3% Triton X-100, then overnight at 4°C with rabbit anti-Smo (1:500; ab38686; Abcam), rabbit anti-caspase-3 cleaved (1:500; 9661S; Cell Signaling Technology), chicken anti-MAP2 (1:5000; ab5392; Abcam), mouse anti-NeuN (1:1000; MAB377; Chemicon), rabbit anti-FoxP2 (1:4000; ab16046; Abcam) or mouse anti-synaptophysin (1:1000; MAB5258; Chemicon). Sections were rinsed in PBS and incubated for 2 h with the corresponding Alexa488- (1:1000; FluoProbes), Cy5- or Cy3-conjugated (1:1000; Chemicon) secondary antibodies diluted in PBS). DAPI (Vector Laboratories H-1200) was applied to stain nuclei. Control tissues used to determine the level of nonspecific staining included tissues incubated without primary antibody. Sequential acquisition of immunoreactivity of GFP-positive pyramidal-like cells was performed using a laser scanning confocal microscope (Zeiss LSM 510 Meta) with a 40× or 63× oil-immersion objective. For each experimental condition, a semi-quantitative analysis of synaptophysin (Syn)-labeled area fraction per field was measured and reported relative to the area fraction of pixels positive for MAP2 staining. The surface areas of NeuN immuno-positive neurons expressing GFP or Smo mutants were measured manually using the Freehands selection tool in ImageJ. For neuronal migration, electroporated rat brains were removed at E20 and fixed in Antigenfix (Diapath) for 24 h before they were included in agar and sectioned (70 µm) using a vibratome (Leica VT 100 S). Analysis was performed as previously described (Elias et al., 2007), with an automated subdivision into regions of equal length, from the ventricle to the surface [i.e. ventricular/subventricular zones (VZ/SVZ), intermediate zone (IZ) and cortical plate (CP)]. In each set of images, laser light levels and detector gain and offset were adjusted to avoid any saturated levels. Optical sections were digitized (1024×1024 pixels) and processed using ImageJ software. All of the images were analyzed blind.

### Relative quantitative expression of mRNA transcripts

Expressions of *Gli1* and *Ptch1* mRNA in the electroporated regions of the somatosensory cortex were measured using real-time RT-qPCR. Total RNA was isolated from cerebral cortices (P15 rats) using a Mini RNeasy kit (Qiagen) then converted to cDNA using 1 µg RNA and a QuantiTect Reverse Transcription kit (Qiagen) according to the manufacturer's instructions. Single-cell gene expression was performed on primary hippocampal neurons transfected with a mixture of constructs encoding mCherry (mock), Smo SA0-5, Smo-ΔN or Smo-CA with or without GANT61 (10 µM) for 48 h. Whole-cell configuration using patch pipettes was used to harvest the cytosol from 15 transfected neurons per condition, which were converted immediately to cDNA as described above. PCR was carried out with a LightCycler 480 SYBR Green I Master (Roche Applied Science) with 1 µL cDNA using the following oligonucleotides (QuantiTect Primer Assays, Qiagen): Gli1 (Gli1; QT01290324), Ptch1 (QT01579669), KCC2 (Slc12a5; QT00145327) and glyceraldehyde-3-phosphate dehydrogenase (GAPDH; QT001199633). Relative mRNA values were calculated using LC480 software with GAPDH as the housekeeping gene. PCR was performed in triplicate.

### Sonic hedgehog protein immunoassay

Cortical tissues from electroporated rats at the indicated age were homogenized in RIPA buffer [150 mM NaCl, 1% Triton X-100, 0.1% SDS, 50 mM Tris-HCl, pH 8, containing protease inhibitors (Complete Mini; Roche)]. Lysates were centrifuged (5000 ***g*** for 5 min at 4°C). Loading was 200 µg of protein, as determined using a modified Bradford reaction (Bio-Rad Laboratories). Quantification of Shh was performed using a Rat Shh ELISA Kit (FineTest, Wuhan Fine Biotech Co. Ltd., China) in the concentrated solutions following the manufacturer's protocol. Experiments and analysis were performed blind.

### Slice preparation and electrophysiological recordings

Electroporated cortical regions of P14 rats were identified using an atlas of the developing rat brain (Khazipov et al., 2015). Electroporated cortical regions of P20 and P30 rats were identified using the Paxinos and Watson rat brain atlas (Paxinos and Watson, 2005). Based on these two atlases, the somatosensory regions were prepared for acute slice experiments from P14, P20 and P30 rats in ice-cold (2–4°C) oxygenated modified artificial cerebrospinal fluid (ACSF) (0.5 mM CaCl_2_ and 7 mM MgSO_4_; NaCl replaced by an equimolar concentration of choline). Slices (300-µm thick) were cut with a Vibratome (VT1000E; Leica, Nussloch, Germany) and kept at room temperature (25°C) for at least one hour before recording in oxygenated normal ACSF containing (in mM): 126 NaCl, 3.5 KCl, 2 CaCl_2_, 1.3 MgCl_2_, 1.2 NaH_2_PO_4_, 25 NaHCO_3_ and 11 glucose, pH 7.4 equilibrated with 95% O_2_ and 5% CO_2_. Slices were then transferred to a submerged recording chamber perfused with ACSF (3 ml/min) at 34°C.

### Spiking activity and data analysis

Extracellular field potential recordings were performed from P14, P20 and P30 electroporated somatosensory cortical slices. Extracellular tungsten electrodes of 50 µm diameter (California Fine Wire, Grover Beach, CA, USA) were positioned in the V/VI cortical pyramidal cell layer of the transfected area to record the multiunit activity (MUA). Field potentials were recorded using a DAM80 Amplifier (World Precision Instruments, Sarasota, FL, USA) using a 1–3 Hz bandpass filter and were analyzed off-line with the Axon package MiniAnalysis program (Jaejin Software, Leonia, NJ, USA). To determine the developmental changes in GABA_A_ signaling, we used isoguvacine, a potent and selective GABA_A_ receptor agonist (Krogsgaard-Larsen and Johnston, 1978). We determined the effect of isoguvacine on MUA as a ratio of the spiking frequency at the peak of the isoguvacine response to the spiking frequency in control.

### Cl-Sensor fluorescence recordings from brain slices

To perform non-invasive monitoring of neuronal intracellular chloride concentration ([Cl^−^]_i_), we used a ratiometric genetically-encoded Cl^−^-sensitive probe called Cl-Sensor (Markova et al., 2008; Waseem et al., 2010) that was co-expressed in the cells of interest together with other constructs as described above. The acquisition of fluorescence images was performed using a customized imaging setup with consecutive cell excitation at 430 and 500 nm and emission at 480 and 540 nm. The frequency of acquisition was 0.05 Hz. The duration of excitation was selected for each cell type and was selected to avoid use-dependent bleaching of the signal (Friedel et al., 2013). Results are expressed as fluorescence ratio measured at 430 and 500 nm excitation wavelengths (R_430/500_). Experiments were performed on acute cortical slices from *in utero* electroporated rat pups on postnatal days P10 and P30. Individual slices were transferred to a specially designed recording chamber where they were fully submerged and superfused with oxygenated ACSF complemented with 1 µM tetrodotoxin, 0.3 µM strychnine and 10 µM NBQX to prevent spontaneous neuronal activity and noncontrolled [Cl^−^]_i_ changes at 30–32°C at a rate of 2–3 ml/min. The applications of the ACSF solution containing isoguvacine (30 µM) or KCl (25 mM)+isoguvacine (30 µM) were performed with a perfusion system.

### Seizure induction with pentylenetetrazol

To evaluate the susceptibility to seizures in control rats (GFP-transfected brains), and in Smo-CA- and Smo-ΔN-expressing animals at P30, pentylenetetrazol (PTZ; 25 mg/kg; Sigma) was administered via intraperitoneal injections every 10 min until generalized seizures occurred. Rats were placed in a plexiglass cage, and the time to the onset of the generalized seizure was measured by observation. There was no significant difference in weight and sex between groups of animals. Experiments were performed blind, and the electroporated region was verified after PTZ induction. Rats with a a very local electroporated cortical area of GFP fluorescence or fluorescence in other brain regions than somatosensory cortex were excluded.

### Primary cultures and transfection of rat hippocampal neurons

Neurons from 18-day-old rat embryos were dissected and dissociated using trypsin and plated at a density of 70,000 cells cm^−2^ in minimal essential medium (MEM) supplemented with 10% NU serum (BD Biosciences, Le Pont de Claix, France), 0.45% glucose, 1 mM sodium pyruvate, 2 mM glutamine and 10 U ml^−1^ penicillin–streptomycin (Buerli et al., 2007). On days 7, 10 and 13 of culture incubation (DIV, days *in vitro*), half of the medium was changed to MEM with 2% B27 supplement (Invitrogen). For electrophysiology, neuronal cultures were plated on coverslips placed in 35-mm culture dishes. Twelve hours before plating, dishes with coverslips were coated with polyethylenimine (5 mg/ml). Transfection of cultured neurons was performed with 300 µl Opti-MEM medium mixed with 7 µl Lipofectamine 2000 (Invitrogen), 1 µl Magnetofection CombiMag (OZ Biosciences) per µg of DNA and 1.5 µg premixed DNA encoding the constructs of interest. The mixture was incubated for 20 min at room temperature and thereafter distributed dropwise above the neuronal culture. Culture dishes (35-mm) were placed on a magnetic plate (OZ Biosciences) and incubated for 35 min at 37°C, 5% CO_2_. Transfection was terminated by the substitution of 80% of the incubation solution with fresh culture medium. Cells were used in the experiments 48–72 h after transfection. These experiments were based on co-transfection into the same cell of two different pcDNAs encoding a fluorescent marker of transfection (eGFP or mCherry, 0.3 µg), and Smo-related constructs (1.2 µg).

### Gramicidin-perforated patch-clamp recordings

Gramicidin-perforated patch-clamp recordings were performed on primary hippocampal neurons, transfected with a mixture of Smo-related constructs (mCherry, Smo SA0-5, Smo-ΔN and Smo-CA) in the presence or absence of GANT61 (10 µM, 48 h) and with or without KCC2-pH_ext_ (Friedel et al., 2017). Measurements were performed 2 or 3 days after transfection (corresponding to 8 or 9 DIV). Coverslips with transfected neurons were placed onto the inverted microscope and perfused with an external HEPES-buffered solution (HBS: 140 mM NaCl, 2.5 mM KCl, 20 mM HEPES, 20 mM d-glucose, 2.0 mM CaCl_2_, 2.0 mM MgCl_2_, and 0.02 mM bumetanide, pH 7.4). For recording from neurons, external HBS contained 0.5 µM tetrodotoxin and 15 µM bumetanide. The recording micropipettes (5 MΩ) were filled with a solution containing 150 mM KCl, 10 mM HEPES and 20 µg/ml gramicidin A, pH 7.2. Isoguvacine (30 µM) was dissolved in an external solution and focally applied to recorded cells through a micropipette connected to a Picospritzer (General Valve Corporation, pressure 5 p.s.i.). Recordings were performed using an Axopatch-200A amplifier and pCLAMP acquisition software (Molecular Devices) in voltage-clamp mode. Data were low-pass filtered at 2 kHz and acquired at 10 kHz. Isoguvacine responses were recorded at voltages −110, −90, −70 and −50 mV, or at −70, −50, −30 and −10 mV depending on neuron GABA inversion potential. A linear regression was used to calculate the best-fit line of the voltage dependence of the isoguvacine responses.

### Surface immunolabeling on living neurons and analysis of KCC2-pH_ext_ proteins

For immunolabeling of KCC2-pH_ext_ proteins on living neurons, rabbit anti-GFP antibody was dissolved in culture medium applied to neurons for 2 h at 37°C, 5% CO2 (Friedel et al., 2015). Neurons were then rinsed three times at room temperature with HBS, labeled with anti-rabbit Cy3-conjugated antibody for 20 min at 13°C and fixed in Antigenfix (Diapath). To reveal the intracellular pool of live-labeled proteins, cells were permeabilized with 0.3% Triton X-100, blocked by 5% goat serum and incubated for 1 h at room temperature with anti-rabbit Alexa647-conjugated antibody. For visualization of the total pool of overexpressed KCC2-pH_ext_, cells were labeled overnight (4°C) with mouse anti-GFP antibody and for 1 h at room temperature with anti-mouse Alexa488-conjugated antibody. For control of the cell membrane integrity during live-cell immunolabeling, one batch of cultures were routinely transfected with the KCC2-pH_ext_ mutant ΔNTD-KCC2-pH_ext_, which does not incorporate into the plasma membrane (Friedel et al., 2017). A/A-KCC2-pH_ext_, a double phosphomimetic mutant of KCC2 for T906A/T1007A that maintains KCC2 at the cell surface (Friedel et al., 2015), was used as a positive control.

Images of labeled neurons were acquired with an Olympus FluoView 500 confocal microscope [oil-immersion objective 60× (NA 1.4); zoom 1–5]. We randomly selected and focused on a transfected cell by only visualizing Alexa488 fluorescence and then acquired *z*-stack images of Alexa488, Cy3 and Alexa647 fluorochromes. Each *z*-stack included ten planes of 1 µm optical thickness, taken at 0.5 µm distance between planes. The cluster properties and fluorescence intensities of each cell were analyzed with Metamorph software. First, we used the logical ‘NOT’ conversion of pairs of Alexa647 and Cy3 images to isolate in each focal plane the Alexa647 signal that was not overlapping with Cy3 fluorescence restricted to the plasma membrane. This gave rise to additional images reflecting the fluorescence of the internalized pool of labeled clusters, called thereafter ‘NOT-conversion’. Second, the arithmetic summation for each *z*-stack and channel was performed to collect the whole fluorescence of the different signals (Alexa488 signal for total protein fluorescence; Cy3 signal for plasma membrane restricted fluorescence; NOT-conversion signal for internalized restricted fluorescence; and Alexa647 signal for all surface-labeled fluorescence). Third, a binary mask was created for each cell, using the Alexa488 image, to isolate the signal coming from the transfected neuron, and the fluorescence parameters (total fluorescence, single cluster fluorescence as well as density and brightness of clusters) were analyzed for each channel (Alexa488, Cy3, NOT-conversion and Alexa647) in regions overlapping with the binary mask. The analysis parameters were the same for each experiment, and all experiments were performed blind. After analysis, data were normalized to the mean value of cells transfected with KCC2-pHext+GFP.

### Statistical analysis

No statistical methods were used to predetermine sample sizes. To ensure the consistency and reproducibility of our results, we conducted repeated trials in different cell cultures and acute brain slices prepared from at least three different animals for each experimental condition. If not stated otherwise, statistics are presented as the mean±s.d. for normally distributed data and as the median only for non-normally distributed data. Experiments with control and Smo-electroporated animals were processed at the same time to ensure homogeneity of experimental conditions.

## Supplementary Material

Reviewer comments

## References

[JCS247700C1] Aguado F., Carmona M. A., Pozas E., Aguiló A., Martínez-Guijarro F. J., Alcantara S., Borrell V., Yuste R., Ibañez C. F. and Soriano E. (2003). BDNF regulates spontaneous correlated activity at early developmental stages by increasing synaptogenesis and expression of the K^+^/Cl^−^ co-transporter KCC2. *Development* 130, 1267-1280. 10.1242/dev.00351 12588844

[JCS247700C2] Al-Ayadhi L. Y. (2012). Relationship between Sonic hedgehog protein, brain-derived neurotrophic factor and oxidative stress in autism spectrum disorders. *Neurochem. Res.* 37, 394-400. 10.1007/s11064-011-0624-x 21984201 PMC3264868

[JCS247700C3] Álvarez-Buylla A. and Ihrie R. A. (2014). Sonic hedgehog signaling in the postnatal brain. *Semin. Cell Dev. Biol.* 33, 105-111. 10.1016/j.semcdb.2014.05.008 24862855 PMC4130786

[JCS247700C4] Antonelli F., Casciati A. and Pazzaglia S. (2019). Sonic hedgehog signaling controls dentate gyrus patterning and adult neurogenesis in the hippocampus. *Neural Regen Res.* 14, 59-61. 10.4103/1673-5374.243703 30531071 PMC6263010

[JCS247700C5] Araújo G. L. L., Araújo J. A. M., Schroeder T., Tort A. B. L. and Costa M. R. (2014). Sonic hedgehog signaling regulates mode of cell division of early cerebral cortex progenitors and increases astrogliogenesis. *Front. Cell Neurosci.* 8, 77 10.3389/fncel.2014.00077 24653675 PMC3949322

[JCS247700C6] Belgacem Y. H. and Borodinsky L. N. (2011). Sonic hedgehog signaling is decoded by calcium spike activity in the developing spinal cord. *Proc. Natl. Acad. Sci. USA* 108, 4482-4487. 10.1073/pnas.1018217108 21368195 PMC3060219

[JCS247700C7] Belgacem Y. H. and Borodinsky L. N. (2015). Inversion of Sonic hedgehog action on its canonical pathway by electrical activity. *Proc. Natl. Acad. Sci. USA* 112, 4140-4145. 10.1073/pnas.1419690112 25829542 PMC4386408

[JCS247700C8] Belgacem Y. H., Hamilton A. M., Shim S., Spencer K. A. and Borodinsky L. N. (2016). The many hats of sonic hedgehog signaling in nervous system development and disease. *J. Dev. Biol.* 4, 35 10.3390/jdb4040035 PMC5831807 29615598

[JCS247700C9] Ben-Ari Y. (2002). Excitatory actions of gaba during development: the nature of the nurture. *Nat. Rev. Neurosci.* 3, 728-739. 10.1038/nrn920 12209121

[JCS247700C10] Ben-Ari Y. and Holmes G. L. (2005). The multiple facets of γ-aminobutyric acid dysfunction in epilepsy. *Curr. Opin. Neurol.* 18, 141-145. 10.1097/01.wco.0000162855.75391.6a 15791144

[JCS247700C11] Ben-Ari Y., Cherubini E., Corradetti R. and Gaiarsa J. L. (1989). Giant synaptic potentials in immature rat CA3 hippocampal neurones. *J. Physiol. (Lond.)* 416, 303-325. 10.1113/jphysiol.1989.sp017762 2575165 PMC1189216

[JCS247700C12] Ben-Ari Y., Gaiarsa J.-L., Tyzio R. and Khazipov R. (2007). GABA: a pioneer transmitter that excites immature neurons and generates primitive oscillations. *Physiol. Rev.* 87, 1215-1284. 10.1152/physrev.00017.2006 17928584

[JCS247700C13] Beug S. T., Parks R. J., McBride H. M. and Wallace V. A. (2011). Processing-dependent trafficking of Sonic hedgehog to the regulated secretory pathway in neurons. *Mol. Cell. Neurosci.* 46, 583-596. 10.1016/j.mcn.2010.12.009 21182949

[JCS247700C14] Bezard E., Baufreton J., Owens G., Crossman A. R., Dudek H., Taupignon A. and Brotchie J. M. (2003). Sonic hedgehog is a neuromodulator in the adult subthalamic nucleus. *FASEB J.* 17, 2337-2338. 10.1096/fj.03-0291fje 14525941

[JCS247700C15] Bond C. W., Angeloni N., Harrington D., Stupp S. and Podlasek C. A. (2013). Sonic Hedgehog regulates brain-derived neurotrophic factor in normal and regenerating cavernous nerves. *J. Sex. Med.* 10, 730-737. 10.1111/jsm.12030 23237228 PMC3593960

[JCS247700C16] Bovolenta R., Zucchini S., Paradiso B., Rodi D., Merigo F., Navarro Mora G., Osculati F., Berto E., Marconi P., Marzola A. et al. (2010). Hippocampal FGF-2 and BDNF overexpression attenuates epileptogenesis-associated neuroinflammation and reduces spontaneous recurrent seizures. *J. Neuroinflammation* 7, 81 10.1186/1742-2094-7-81 21087489 PMC2993685

[JCS247700C17] Brambilla P., Hardan A., di Nemi S. U., Perez J., Soares J. C. and Barale F. (2003). Brain anatomy and development in autism: review of structural MRI studies. *Brain Res. Bull.* 61, 557-569. 10.1016/j.brainresbull.2003.06.001 14519452

[JCS247700C18] Breunig J. J., Sarkisian M. R., Arellano J. I., Morozov Y. M., Ayoub A. E., Sojitra S., Wang B., Flavell R. A., Rakic P. and Town T. (2008). Primary cilia regulate hippocampal neurogenesis by mediating sonic hedgehog signaling. *Proc. Natl. Acad. Sci. USA* 105, 13127-13132. 10.1073/pnas.0804558105 18728187 PMC2529104

[JCS247700C19] Briscoe J. and Thérond P. P. (2013). The mechanisms of Hedgehog signalling and its roles in development and disease. *Nat. Rev. Mol. Cell Biol.* 14, 416-429. 10.1038/nrm3598 23719536

[JCS247700C20] Buerli T., Pellegrino C., Baer K., Lardi-Studler B., Chudotvorova I., Fritschy J.-M., Medina I. and Fuhrer C. (2007). Efficient transfection of DNA or shRNA vectors into neurons using magnetofection. *Nat. Protoc.* 2, 3090-3101. 10.1038/nprot.2007.445 18079708

[JCS247700C21] Chamma I., Heubl M., Chevy Q., Renner M., Moutkine I., Eugène E., Poncer J. C. and Lévi S. (2013). Activity-dependent regulation of the K/Cl transporter KCC2 membrane diffusion, clustering, and function in hippocampal neurons. *J. Neurosci.* 33, 15488-15503. 10.1523/JNEUROSCI.5889-12.2013 24068817 PMC6618451

[JCS247700C22] Charytoniuk D., Porcel B., Rodríguez Gomez J., Faure H., Ruat M. and Traiffort E. (2002). Sonic Hedgehog signalling in the developing and adult brain. *J. Physiol. Paris* 96, 9-16. 10.1016/S0928-4257(01)00075-4 11755778

[JCS247700C23] Chen Y., Sasai N., Ma G., Yue T., Jia J., Briscoe J. and Jiang J. (2011). Sonic Hedgehog dependent phosphorylation by CK1α and GRK2 is required for ciliary accumulation and activation of smoothened. *PLoS Biol.* 9, e1001083 10.1371/journal.pbio.1001083 21695114 PMC3114773

[JCS247700C24] Chen L., Wan L., Wu Z., Ren W., Huang Y., Qian B. and Wang Y. (2017). KCC2 downregulation facilitates epileptic seizures. *Sci. Rep.* 7, 156 10.1038/s41598-017-00196-7 28279020 PMC5427808

[JCS247700C25] Chen S.-D., Yang J.-L., Hwang W.-C. and Yang D.-I. (2018). Emerging roles of sonic hedgehog in adult neurological diseases: neurogenesis and beyond. *Int. J. Mol. Sci.* 19, 2423 10.3390/ijms19082423 PMC6121355 30115884

[JCS247700C26] Choi J., Ababon M. R., Soliman M., Lin Y., Brzustowicz L. M., Matteson P. G. and Millonig J. H. (2014). Autism associated gene, engrailed2, and flanking gene levels are altered in post-mortem cerebellum. *PLoS ONE* 9, e87208 10.1371/journal.pone.0087208 24520327 PMC3919719

[JCS247700C27] Choudhry Z., Rikani A. A., Choudhry A. M., Tariq S., Zakaria F., Asghar M. W., Sarfraz M. K., Haider K., Shafiq A. A. and Mobassarah N. J. (2014). Sonic hedgehog signalling pathway: a complex network. *Ann. Neurosci.* 21, 28-31. 10.5214/ans.0972.7531.210109 25206052 PMC4117150

[JCS247700C28] Côme E., Marques X., Poncer J. C. and Lévi S. (2019). KCC2 membrane diffusion tunes neuronal chloride homeostasis. *Neuropharmacology* 169, 107571 10.1016/j.neuropharm.2019.03.014 30871970

[JCS247700C29] de Los Heros P., Alessi D. R., Gourlay R., Campbell D. G., Deak M., Macartney T. J., Kahle K. T. and Zhang J. (2014). The WNK-regulated SPAK/OSR1 kinases directly phosphorylate and inhibit the K+-Cl− co-transporters. *Biochem. J.* 458, 559-573. 10.1042/BJ20131478 24393035 PMC3940040

[JCS247700C30] Delmotte Q., Diabira D., Belaidouni Y., Hamze M., Kochmann M., Montheil A., Gaiarsa J.-L., Porcher C. and Belgacem Y. H. (2020). Sonic hedghog signaling agonist (SAG) triggers BDNF secretion and promotes the maturation of GABAergic networks in the postanatal rat hippocampus. *Front. Cell. Neurosci.* 14, 98 10.3389/fncel.2020.00098 32425757 PMC7212340

[JCS247700C31] Dumon C., Diabira D., Chudotvorova I., Bader F., Sahin S., Zhang J., Porcher C., Wayman G., Medina I. and Gaiarsa J.-L. (2018). The adipocyte hormone leptin sets the emergence of hippocampal inhibition in mice. *Elife* 7, e36726 10.7554/eLife.36726 30106375 PMC6112852

[JCS247700C32] Elias L. A. B., Wang D. D. and Kriegstein A. R. (2007). Gap junction adhesion is necessary for radial migration in the neocortex. *Nature* 448, 901-907. 10.1038/nature06063 17713529

[JCS247700C33] Falcicchia C., Paolone G., Emerich D. F., Lovisari F., Bell W. J., Fradet T., Wahlberg L. U. and Simonato M. (2018). Seizure-suppressant and neuroprotective effects of encapsulated BDNF-producing cells in a rat model of temporal lobe epilepsy. *Mol. Ther. Methods Clin. Dev.* 9, 211-224. 10.1016/j.omtm.2018.03.001 29766029 PMC5948312

[JCS247700C34] Fang M., Lu Y., Chen G.-J., Shen L., Pan Y.-M. and Wang X.-F. (2011). Increased expression of sonic hedgehog in temporal lobe epileptic foci in humans and experimental rats. *Neuroscience* 182, 62-70. 10.1016/j.neuroscience.2011.02.060 21376786

[JCS247700C35] Feng S., Ma S., Jia C., Su Y., Yang S., Zhou K., Liu Y., Cheng J., Lu D., Fan L. et al. (2016). Sonic hedgehog is a regulator of extracellular glutamate levels and epilepsy. *EMBO Rep.* 17, 682-694. 10.15252/embr.201541569 27113760 PMC5341526

[JCS247700C36] Friedel P., Bregestovski P. and Medina I. (2013). Improved method for efficient imaging of intracellular Cl(−) with Cl-Sensor using conventional fluorescence setup. *Front. Mol. Neurosci.* 6, 7 10.3389/fnmol.2013.00007 23596389 PMC3622059

[JCS247700C37] Friedel P., Kahle K. T., Zhang J., Hertz N., Pisella L. I., Buhler E., Schaller F., Duan J., Khanna A. R., Bishop P. N. et al. (2015). WNK1-regulated inhibitory phosphorylation of the KCC2 cotransporter maintains the depolarizing action of GABA in immature neurons. *Sci. Signal.* 8, ra65 10.1126/scisignal.aaa0354 26126716

[JCS247700C38] Friedel P., Ludwig A., Pellegrino C., Agez M., Jawhari A., Rivera C. and Medina I. (2017). A novel view on the role of intracellular tails in surface delivery of the potassium-chloride cotransporter KCC2. *eNeuro* 4, ENEURO.0055-17.2017 10.1523/ENEURO.0055-17.2017 PMC5520751 28785725

[JCS247700C39] Halepoto D. M., Bashir S., Zeina R. and Al-Ayadhi L. Y. (2015). Correlation between hedgehog (Hh) protein family and brain-derived neurotrophic factor (BDNF) in autism spectrum disorder (ASD). *J. Coll. Physicians Surg. Pak.* 25, 882-885. 26691363

[JCS247700C40] Hammond R., Blaess S. and Abeliovich A. (2009). Sonic hedgehog is a chemoattractant for midbrain dopaminergic axons. *PLoS ONE* 4, e7007 10.1371/journal.pone.0007007 19774071 PMC2742719

[JCS247700C41] Harwell C. C., Parker P. R. L., Gee S. M., Okada A., McConnell S. K., Kreitzer A. C. and Kriegstein A. R. (2012). Sonic hedgehog expression in corticofugal projection neurons directs cortical microcircuit formation. *Neuron* 73, 1116-1126. 10.1016/j.neuron.2012.02.009 22445340 PMC3551478

[JCS247700C42] Hildebrand M. S., Griffin N. G., Damiano J. A., Cops E. J., Burgess R., Ozturk E., Jones N. C., Leventer R. J., Freeman J. L., Harvey A. S. et al. (2016). Mutations of the sonic hedgehog pathway underlie hypothalamic hamartoma with Gelastic epilepsy. *Am. J. Hum. Genet.* 99, 423-429. 10.1016/j.ajhg.2016.05.031 27453577 PMC4974069

[JCS247700C43] Hill S. A., Blaeser A. S., Coley A. A., Xie Y., Shepard K. A., Harwell C. C., Gao W.-J. and Garcia A. D. R. (2019). Sonic hedgehog signaling in astrocytes mediates cell type-specific synaptic organization. *Elife* 8, e45545 10.7554/eLife.45545.029 31194676 PMC6629371

[JCS247700C44] Inoue K., Furukawa T., Kumada T., Yamada J., Wang T., Inoue R. and Fukuda A. (2012). Taurine inhibits K+-Cl− cotransporter KCC2 to regulate embryonic Cl- homeostasis via with-no-lysine (WNK) protein kinase signaling pathway. *J. Biol. Chem.* 287, 20839-20850. 10.1074/jbc.M111.319418 22544747 PMC3375508

[JCS247700C45] Jacob L. and Lum L. (2007). Deconstructing the hedgehog pathway in development and disease. *Science* 318, 66-68. 10.1126/science.1147314 17916724 PMC3791603

[JCS247700C46] Jeong J., Mao J., Tenzen T., Kottmann A. H. and McMahon A. P. (2004). Hedgehog signaling in the neural crest cells regulates the patterning and growth of facial primordia. *Genes Dev.* 18, 937-951. 10.1101/gad.1190304 15107405 PMC395852

[JCS247700C47] Kahle K. T. and Delpire E. (2016). Kinase-KCC2 coupling: Cl- rheostasis, disease susceptibility, therapeutic target. *J. Neurophysiol.* 115, 8-18. 10.1152/jn.00865.2015 26510764 PMC4760510

[JCS247700C48] Kahle K. T., Deeb T. Z., Puskarjov M., Silayeva L., Liang B., Kaila K. and Moss S. J. (2013). Modulation of neuronal activity by phosphorylation of the K-Cl cotransporter KCC2. *Trends Neurosci.* 36, 726-737. 10.1016/j.tins.2013.08.006 24139641 PMC4381966

[JCS247700C49] Kahle K. T., Merner N. D., Friedel P., Silayeva L., Liang B., Khanna A., Shang Y., Lachance-Touchette P., Bourassa C., Levert A. et al. (2014). Genetically encoded impairment of neuronal KCC2 cotransporter function in human idiopathic generalized epilepsy. *EMBO Rep.* 15, 766-774. 10.15252/embr.201438840 24928908 PMC4196980

[JCS247700C50] Kelsch W., Hormuzdi S., Straube E., Lewen A., Monyer H. and Misgeld U. (2001). Insulin-like growth factor 1 and a cytosolic tyrosine kinase activate chloride outward transport during maturation of hippocampal neurons. *J. Neurosci.* 21, 8339-8347. 10.1523/JNEUROSCI.21-21-08339.2001 11606621 PMC6762818

[JCS247700C51] Khazipov R., Zaynutdinova D., Ogievetsky E., Valeeva G., Mitrukhina O., Manent J.-B. and Represa A. (2015). Atlas of the postnatal rat brain in stereotaxic coordinates. *Front. Neuroanat.* 9, 161 10.3389/fnana.2015.00161 26778970 PMC4688355

[JCS247700C52] Khirug S., Huttu K., Ludwig A., Smirnov S., Voipio J., Rivera C., Kaila K. and Khiroug L. (2005). Distinct properties of functional KCC2 expression in immature mouse hippocampal neurons in culture and in acute slices. *Eur. J. Neurosci.* 21, 899-904. 10.1111/j.1460-9568.2005.03886.x 15787696

[JCS247700C53] Kilb W., Kirischuk S. and Luhmann H. J. (2013). Role of tonic GABAergic currents during pre- and early postnatal rodent development. *Front. Neural. Circuits* 7, 139 10.3389/fncir.2013.00139 24027498 PMC3760143

[JCS247700C54] Kim J., Kato M. and Beachy P. A. (2009). Gli2 trafficking links Hedgehog-dependent activation of Smoothened in the primary cilium to transcriptional activation in the nucleus. *Proc. Natl. Acad. Sci. USA* 106, 21666-21671. 10.1073/pnas.0912180106 19996169 PMC2790365

[JCS247700C55] Kirmse K., Kummer M., Kovalchuk Y., Witte O. W., Garaschuk O. and Holthoff K. (2015). GABA depolarizes immature neurons and inhibits network activity in the neonatal neocortex in vivo. *Nat. Commun.* 6, 7750 10.1038/ncomms8750 26177896

[JCS247700C56] Klioueva I. A., van Luijtelaar E. L., Chepurnova N. E. and Chepurnov S. A. (2001). PTZ-induced seizures in rats: effects of age and strain. *Physiol. Behav.* 72, 421-426. 10.1016/S0031-9384(00)00425-X 11274687

[JCS247700C57] Kriegstein A. R. and Noctor S. C. (2004). Patterns of neuronal migration in the embryonic cortex. *Trends Neurosci.* 27, 392-399. 10.1016/j.tins.2004.05.001 15219738

[JCS247700C58] Krogsgaard-Larsen P. and Johnston G. A. (1978). Structure-activity studies on the inhibition of GABA binding to rat brain membranes by muscimol and related compounds. *J. Neurochem.* 30, 1377-1382. 10.1111/j.1471-4159.1978.tb10469.x 670980

[JCS247700C59] Kuzirian M. S. and Paradis S. (2011). Emerging themes in GABAergic synapse development. *Prog. Neurobiol.* 95, 68-87. 10.1016/j.pneurobio.2011.07.002 21798307 PMC3166545

[JCS247700C60] Lauth M., Bergström A., Shimokawa T. and Toftgård R. (2007). Inhibition of GLI-mediated transcription and tumor cell growth by small-molecule antagonists. *Proc. Natl. Acad. Sci. USA* 104, 8455-8460. 10.1073/pnas.0609699104 17494766 PMC1866313

[JCS247700C61] Lee H. H. C., Walker J. A., Williams J. R., Goodier R. J., Payne J. A. and Moss S. J. (2007). Direct protein kinase C-dependent phosphorylation regulates the cell surface stability and activity of the potassium chloride cotransporter KCC2. *J. Biol. Chem.* 282, 29777-29784. 10.1074/jbc.M705053200 17693402

[JCS247700C62] Lee H. H. C., Deeb T. Z., Walker J. A., Davies P. A. and Moss S. J. (2011). NMDA receptor activity downregulates KCC2 resulting in depolarizing GABAA receptor-mediated currents. *Nat. Neurosci.* 14, 736-743. 10.1038/nn.2806 21532577 PMC3102766

[JCS247700C63] Leonzino M., Busnelli M., Antonucci F., Verderio C., Mazzanti M. and Chini B. (2016). The timing of the excitatory-to-inhibitory GABA switch is regulated by the oxytocin receptor via KCC2. *Cell Rep* 15, 96-103. 10.1016/j.celrep.2016.03.013 27052180 PMC4826440

[JCS247700C64] Logue S. E. and Martin S. J. (2008). Caspase activation cascades in apoptosis. *Biochem. Soc. Trans.* 36, 1-9. 10.1042/BST0360001 18208375

[JCS247700C65] Ludwig A., Uvarov P., Soni S., Thomas-Crusells J., Airaksinen M. S. and Rivera C. (2011). Early growth response 4 mediates BDNF induction of potassium chloride cotransporter 2 transcription. *J. Neurosci.* 31, 644-649. 10.1523/JNEUROSCI.2006-10.2011 21228173 PMC6623457

[JCS247700C66] Lüttjohann A., Fabene P. F. and van Luijtelaar G. (2009). A revised Racine's scale for PTZ-induced seizures in rats. *Physiol. Behav.* 98, 579-586. 10.1016/j.physbeh.2009.09.005 19772866

[JCS247700C67] Maisonpierre P. C., Belluscio L., Friedman B., Alderson R. F., Wiegand S. J., Furth M. E., Lindsay R. M. and Yancopoulos G. D. (1990). NT-3, BDNF, and NGF in the developing rat nervous system: parallel as well as reciprocal patterns of expression. *Neuron* 5, 501-509. 10.1016/0896-6273(90)90089-X 1688327

[JCS247700C68] Markova O., Mukhtarov M., Real E., Jacob Y. and Bregestovski P. (2008). Genetically encoded chloride indicator with improved sensitivity. *J. Neurosci. Methods* 170, 67-76. 10.1016/j.jneumeth.2007.12.016 18279971

[JCS247700C69] Medina I., Friedel P., Rivera C., Kahle K. T., Kourdougli N., Uvarov P. and Pellegrino C. (2014). Current view on the functional regulation of the neuronal K^+−^Cl^−^ cotransporter KCC2. *Front. Cell Neurosci.* 8, 27 10.3389/fncel.2014.00027 24567703 PMC3915100

[JCS247700C70] Memi F., Zecevic N. and Radonjić N. (2018). Multiple roles of Sonic Hedgehog in the developing human cortex are suggested by its widespread distribution. *Brain Struct. Funct.* 223, 2361-2375. 10.1007/s00429-018-1621-5 29492654 PMC5968052

[JCS247700C71] Menshanov P. N., Lanshakov D. A. and Dygalo N. N. (2015). proBDNF is a major product of bdnf gene expressed in the perinatal rat cortex. *Physiol. Res.* 64, 925-934. 10.33549/physiolres.932996 26047381

[JCS247700C72] Mitchell N., Petralia R. S., Currier D. G., Wang Y.-X., Kim A., Mattson M. P. and Yao P. J. (2012). Sonic hedgehog regulates presynaptic terminal size, ultrastructure and function in hippocampal neurons. *J. Cell. Sci.* 125, 4207-4213. 10.1242/jcs.105080 22641692 PMC3516435

[JCS247700C73] Moore Y. E., Kelley M. R., Brandon N. J., Deeb T. Z. and Moss S. J. (2017). Seizing control of KCC2: a new therapeutic target for epilepsy. *Trends Neurosci.* 40, 555-571. 10.1016/j.tins.2017.06.008 28803659

[JCS247700C74] Moore Y. E., Deeb T. Z., Chadchankar H., Brandon N. J. and Moss S. J. (2018). Potentiating KCC2 activity is sufficient to limit the onset and severity of seizures. *Proc. Natl. Acad. Sci. USA* 115, 10166-10171. 10.1073/pnas.1810134115 30224498 PMC6176565

[JCS247700C75] Mueller T. M., Remedies C. E., Haroutunian V. and Meador-Woodruff J. H. (2015). Abnormal subcellular localization of GABAA receptor subunits in schizophrenia brain. *Transl. Psychiatry* 5, e612 10.1038/tp.2015.102 26241350 PMC4564557

[JCS247700C76] Paradiso B., Marconi P., Zucchini S., Berto E., Binaschi A., Bozac A., Buzzi A., Mazzuferi M., Magri E., Navarro Mora G. et al. (2009). Localized delivery of fibroblast growth factor-2 and brain-derived neurotrophic factor reduces spontaneous seizures in an epilepsy model. *Proc. Natl. Acad. Sci. USA* 106, 7191-7196. 10.1073/pnas.0810710106 19366663 PMC2678472

[JCS247700C77] Parra L. M. and Zou Y. (2010). Sonic hedgehog induces response of commissural axons to Semaphorin repulsion during midline crossing. *Nat. Neurosci.* 13, 29-35. 10.1038/nn.2457 19946319

[JCS247700C78] Pascual O., Traiffort E., Baker D. P., Galdes A., Ruat M. and Champagnat J. (2005). Sonic hedgehog signalling in neurons of adult ventrolateral nucleus tractus solitarius. *Eur. J. Neurosci.* 22, 389-396. 10.1111/j.1460-9568.2005.04223.x 16045492

[JCS247700C79] Paxinos G. and Watson C. (2005). *The Rat Brain in Stereotaxic Coordinates*, 7th Edn. Cambridge, MA: Academic Press

[JCS247700C80] Petralia R. S., Schwartz C. M., Wang Y.-X., Mattson M. P. and Yao P. J. (2011). Subcellular localization of Patched and Smoothened, the receptors for Sonic hedgehog signaling, in the hippocampal neuron. *J. Comp. Neurol.* 519, 3684-3699. 10.1002/cne.22681 21618238 PMC3196849

[JCS247700C81] Petralia R. S., Wang Y.-X., Mattson M. P. and Yao P. J. (2012). Subcellular distribution of patched and smoothened in the cerebellar neurons. *Cerebellum* 11, 972-981. 10.1007/s12311-012-0374-6 22477363 PMC3495249

[JCS247700C82] Qin S., Sun D., Zhang C., Tang Y., Zhou F., Zheng K., Tang R. and Zheng Y. (2019). Downregulation of sonic hedgehog signaling in the hippocampus leads to neuronal apoptosis in high-fat diet-fed mice. *Behav. Brain Res.* 367, 91-100. 10.1016/j.bbr.2019.03.055 30940514

[JCS247700C83] Radonjić N. V., Memi F., Ortega J. A., Glidden N., Zhan H. and Zecevic N. (2016). The role of sonic hedgehog in the specification of human cortical progenitors In Vitro. *Cereb. Cortex* 26, 131-143. 10.1093/cercor/bhu183 25146370 PMC4677972

[JCS247700C84] Radzikinas K., Aven L., Jiang Z., Tran T., Paez-Cortez J., Boppidi K., Lu J., Fine A. and Ai X. (2011). A Shh/miR-206/BDNF cascade coordinates innervation and formation of airway smooth muscle. *J. Neurosci.* 31, 15407-15415. 10.1523/JNEUROSCI.2745-11.2011 22031887 PMC3222097

[JCS247700C85] Riffault B., Kourdougli N., Dumon C., Ferrand N., Buhler E., Schaller F., Chambon C., Rivera C., Gaiarsa J.-L. and Porcher C. (2018). Pro-brain-derived neurotrophic factor (proBDNF)-mediated p75NTR activation promotes depolarizing actions of GABA and increases susceptibility to epileptic seizures. *Cereb. Cortex* 28, 510-527. 10.1093/cercor/bhw385 27913431

[JCS247700C86] Riobo N. A., Saucy B., Dilizio C. and Manning D. R. (2006). Activation of heterotrimeric G proteins by Smoothened. *Proc. Natl. Acad. Sci. USA* 103, 12607-12612. 10.1073/pnas.0600880103 16885213 PMC1567926

[JCS247700C87] Rivell A., Petralia R. S., Wang Y.-X., Clawson E., Moehl K., Mattson M. P. and Yao P. J. (2019). Sonic hedgehog expression in the postnatal brain. *Biol. Open* 8, bio040592 10.1242/bio.040592 30837226 PMC6451348

[JCS247700C88] Rivera C., Voipio J., Payne J. A., Ruusuvuori E., Lahtinen H., Lamsa K., Pirvola U., Saarma M. and Kaila K. (1999). The K+/Cl− co-transporter KCC2 renders GABA hyperpolarizing during neuronal maturation. *Nature* 397, 251-255. 10.1038/16697 9930699

[JCS247700C89] Ruat M., Roudaut H., Ferent J. and Traiffort E. (2012). Hedgehog trafficking, cilia and brain functions. *Differentiation* 83, S97-S104. 10.1016/j.diff.2011.11.011 22169886

[JCS247700C90] Saitsu H., Sonoda M., Higashijima T., Shirozu H., Masuda H., Tohyama J., Kato M., Nakashima M., Tsurusaki Y., Mizuguchi T. et al. (2016). Somatic mutations in GLI3 and OFD1 involved in sonic hedgehog signaling cause hypothalamic hamartoma. *Ann. Clin. Transl. Neurol.* 3, 356-365. 10.1002/acn3.300 27231705 PMC4863748

[JCS247700C91] Sawano E., Takahashi M., Negishi T. and Tashiro T. (2013). Thyroid hormone-dependent development of the GABAergic pre- and post-synaptic components in the rat hippocampus. *Int. J. Dev. Neurosci.* 31, 751-761. 10.1016/j.ijdevneu.2013.09.007 24076339

[JCS247700C92] Sernagor E., Chabrol F., Bony G. and Cancedda L. (2010). GABAergic control of neurite outgrowth and remodeling during development and adult neurogenesis: general rules and differences in diverse systems. *Front. Cell Neurosci.* 4, 11 10.3389/fncel.2010.00011 20428495 PMC2859806

[JCS247700C93] Silayeva L., Deeb T. Z., Hines R. M., Kelley M. R., Munoz M. B., Lee H. H. C., Brandon N. J., Dunlop J., Maguire J., Davies P. A. et al. (2015). KCC2 activity is critical in limiting the onset and severity of status epilepticus. *Proc. Natl. Acad. Sci. USA* 112, 3523-3528. 10.1073/pnas.1415126112 25733865 PMC4371976

[JCS247700C94] Spoljaric A., Seja P., Spoljaric I., Virtanen M. A., Lindfors J., Uvarov P., Summanen M., Crow A. K., Hsueh B., Puskarjov M. et al. (2017). Vasopressin excites interneurons to suppress hippocampal network activity across a broad span of brain maturity at birth. *Proc. Natl. Acad. Sci. USA* 114, E10819-E10828. 10.1073/pnas.1717337114 29183979 PMC5740624

[JCS247700C95] Su Y., Yuan Y., Feng S., Ma S. and Wang Y. (2017). High frequency stimulation induces sonic hedgehog release from hippocampal neurons. *Sci. Rep.* 7, 43865 10.1038/srep43865 28262835 PMC5338313

[JCS247700C96] Traiffort E., Angot E. and Ruat M. (2010). Sonic Hedgehog signaling in the mammalian brain. *J. Neurochem.* 113, 576-590. 10.1111/j.1471-4159.2010.06642.x 20218977

[JCS247700C97] Tyzio R., Cossart R., Khalilov I., Minlebaev M., Hübner C. A., Represa A., Ben-Ari Y. and Khazipov R. (2006). Maternal oxytocin triggers a transient inhibitory switch in GABA signaling in the fetal brain during delivery. *Science* 314, 1788-1792. 10.1126/science.1133212 17170309

[JCS247700C98] Ung D. C., Iacono G., Méziane H., Blanchard E., Papon M.-A., Selten M., van Rhijn J.-R., Montjean R., Rucci J., Martin S. et al. (2018). Ptchd1 deficiency induces excitatory synaptic and cognitive dysfunctions in mouse. *Mol. Psychiatry* 23, 1356-1367. 10.1038/mp.2017.39 28416808 PMC5984103

[JCS247700C99] Vernadakis A. and Woodbury D. M. (1969). The developing animal as a model. *Epilepsia* 10, 163-178. 10.1111/j.1528-1157.1969.tb03841.x 4308259

[JCS247700C100] Wang D. D. and Kriegstein A. R. (2009). Defining the role of GABA in cortical development. *J. Physiol. (Lond.)* 587, 1873-1879. 10.1113/jphysiol.2008.167635 19153158 PMC2689328

[JCS247700C101] Wang B., Zhang Y., Dong H., Gong S., Wei B., Luo M., Wang H., Wu X., Liu W., Xu X. et al. (2018). Loss of Tctn3 causes neuronal apoptosis and neural tube defects in mice. *Cell Death Dis* 9, 520 10.1038/s41419-018-0563-4 29725084 PMC5938703

[JCS247700C102] Waseem T., Mukhtarov M., Buldakova S., Medina I. and Bregestovski P. (2010). Genetically encoded Cl-Sensor as a tool for monitoring of Cl-dependent processes in small neuronal compartments. *J. Neurosci. Methods* 193, 14-23. 10.1016/j.jneumeth.2010.08.002 20705097

[JCS247700C103] Wu C. and Sun D. (2015). GABA receptors in brain development, function, and injury. *Metab. Brain Dis.* 30, 367-379. 10.1007/s11011-014-9560-1 24820774 PMC4231020

[JCS247700C104] Yamada J., Okabe A., Toyoda H., Kilb W., Luhmann H. J. and Fukuda A. (2004). Cl- uptake promoting depolarizing GABA actions in immature rat neocortical neurones is mediated by NKCC1. *J. Physiol. (Lond.)* 557, 829-841. 10.1113/jphysiol.2004.062471 15090604 PMC1665166

[JCS247700C105] Yao P. J., Petralia R. S., Ott C., Wang Y.-X., Lippincott-Schwartz J. and Mattson M. P. (2015). Dendrosomatic sonic hedgehog signaling in hippocampal neurons regulates axon elongation. *J. Neurosci.* 35, 16126-16141. 10.1523/JNEUROSCI.1360-15.2015 26658865 PMC4682780

[JCS247700C106] Yao P. J., Petralia R. S. and Mattson M. P. (2016). Sonic hedgehog signaling and hippocampal neuroplasticity. *Trends Neurosci.* 39, 840-850. 10.1016/j.tins.2016.10.001 27865563 PMC5148655

[JCS247700C107] Zhang X. M., Ramalho-Santos M. and McMahon A. P. (2001). Smoothened mutants reveal redundant roles for Shh and Ihh signaling including regulation of L/R asymmetry by the mouse node. *Cell* 105, 781-792. 10.1016/S0092-8674(01)00385-3 11440720

[JCS247700C108] Zuñiga N. R. and Stoeckli E. T. (2017). Sonic -‘Jack-of-All-Trades’ in neural circuit formation. *J. Dev. Biol.* 5, 2 10.3390/jdb5010002 PMC5831768 29615560

