# Supplementary material for: Smoothened receptor signaling regulates the developmental shift of GABA polarity in rat somatosensory cortex
Source: J Cell Sci. 2020 Oct 23;133(20):jcs247700. doi: 10.1242/jcs.247700 (PMC7595691; doi:10.1242/jcs.247700)
Supplement: Reviewer comments [file joces-133-247700_review_history.pdf]

Smoothened receptor signaling regulates the developmental shift of GABA polarity in rat somatosensory cortex

Quentin Delmotte, Mira Hamze, Igor Medina, Emmanuelle Buhler, Jinwei Zhang, Yesser H. Belgacem and Christophe Porcher
DOI: 10.1242/jcs.247700

Editor: Giampietro Schiavo

Review timeline

Original submission:	17 April 2020
Editorial decision:	3 June 2020
First revision received:	12 August 2020
Accepted:	12 September 2020

Original submission

First decision letter

MS ID#: JOCES/2020/247700

MS TITLE: Smoothened receptor signaling regulates the developmental shift of GABA polarity in rat somatosensory cortex

AUTHORS: Quentin Delmotte, Igor Medina, Mira Hamze, Emmanuelle Buhler, Jinwei Zhang, Yesser H Belgacem, and Christophe Porcher
ARTICLE TYPE: Research Article

We have now reached a decision on the above manuscript.

To see the reviewers' reports and a copy of this decision letter, please go to: https://submit-jcs.biologists.org and click on the 'Manuscripts with Decisions' queue in the Author Area. (Corresponding author only has access to reviews.)

As you will see, the reviewers raise a number of substantial criticisms that prevent me from accepting the paper at this stage. They suggest, however, that a revised version might prove acceptable, if you can address their concerns. If you think that you can deal satisfactorily with the criticisms on revision, I would be pleased to see a revised manuscript. We would then return it to the reviewers.

We are aware that you may currently be unable to access the lab to undertake experimental revisions. If it would be helpful, we encourage you to contact us to discuss your revision in greater detail. Please send us a point-by-point response indicating where you are able to address concerns raised (either experimentally or by changes to the text) and where you will not be able to do so within the normal timeframe of a revision. We will then provide further guidance. Please also note that we are happy to extend revision timeframes as necessary.

Please ensure that you clearly highlight all changes made in the revised manuscript. Please avoid using 'Tracked changes' in Word files as these are lost in PDF conversion.

I should be grateful if you would also provide a point-by-point response detailing how you have dealt with the points raised by the reviewers in the 'Response to Reviewers' box. Please attend to

© 2020. Published by The Company of Biologists under the terms of the Creative Commons Attribution License (https://creativecommons.org/licenses/by/4.0/).

all of the reviewers' comments. If you do not agree with any of their criticisms or suggestions please explain clearly why this is so.

Reviewer 1

Advance summary and potential significance to field

In this manuscript the authors describe the consequence of electroporation of activated and inhibitory forms of Smo on the maturation of GABA neurons. It appears that Smo activation results in an earlier appearance of more mature forms of GABA neurons.

Comments for the author

In this manuscript the authors describe the consequence of electroporation of activated and inhibitory forms of Smo on the maturation of GABA neurons. It appears that Smo activation results in an earlier appearance of more mature forms of GABA neurons.

The delivery of constructs via electroporation is convincing and has the advantage of reaching predominantly cells lining the ventricles thus limiting delivery to fewer cell types.

A main concern is the absence of a very obvious control, the use of Wt Smo. This is critical as the high level of expression that is sometimes achieved by electroporation using constructs with strong promoters leads to maturation and localization issues, that should be controlled for using Wt Smo. The demonstration that the transcriptional Hh pathway is affected using Gli and Ptch1 RNA levels is not very convincing and might not demonstrate the barely significant difference when compared to this appropriate control.

Given the marginal induction/repression of the Hh response pathway after electroporation, a further demonstration of the involvement of the transcription Hh response pathway would be the use of blocking and activated forms of Gli. In particular the truncated forms of Gli3 are much more powerful dominant negatives that the inhibitory forms of Smo.

Although mentioned several times, nowhere in this paper is it demonstrated that the effects on GABA maturation are mediated by Shh, only that a possible upregulation of the Hh response downstream of Smo has an effect on GABA neuron maturation. For that, something like 5E1 blocking experiments have to be performed. It would be a good addition to the paper to demonstrate that Shh electroporation in general gives the same phenotype as electroporation of activated Smo, and it would help to demonstrate that Shh is involved the GABA neuron maturation.

Reviewer 2

Advance summary and potential significance to field

The study of Delmotte et al. elegantly shows a role of the Hedgehog core transducer Smoothened (SMO) in maturation of the GABAergic properties in the somatosensory cortex in rats, which is essential to prevent seizures in adult animals. It shows a novel function of SMO in promoting the stabilisation of the K+/Cl- KCC2 channel at the plasma membrane, to mediate a developmental shift of GABAa receptors form depolarising to hyperpolarising.

Comments for the author

The study combines in utero electroporation of rat embryos to introduce constitutively active or dominant negative SMO; together with expression in primary neuron cultures to show that SMO promotes stabilisation of the K+/Cl- KCC2 channel at the plasma membrane, to mediate a developmental shift of GABAa receptors form depolarising to hyperpolarising. These findings are convincing and well-controlled however they generate key questions on the specific function of SMO in the process and the role of Hh ligands in controlling SMO's activity:

© 2020. Published by The Company of Biologists under the terms of the Creative Commons Attribution License (https://creativecommons.org/licenses/by/4.0/).

1- Is SMO acting through the canonical (GLI-dependent) Hh pathway? Experiments in neuronal cultures could be performed using GLI inhibitors and/or GLI3R to investigate their involvement.

2- a previous report from one of the authors revealed a non-canonical role of SMO in neuronal progenitors Ca++ spikes, through coupling to G inhibitory proteins. Are Gi proteins mediators of SMO in this context?

3- the inactive form of SMO used, called dominant-negative, is a deletion of a portion of the C-tail that has showed inability to activate GLI-dependent transcription and reduces endogenous SMO activity, therefore being proposed as a dominant negative. However, the inhibitory activity of this DN-SMO mutant is far from complete, and its effect on non-canonical HH signalling, in particular on Gi protein activation is unknown.
For example, wild type SMO does not require the C-tail for Gi coupling, but the effect of this partial deletion is unknown. Therefore, it will be necessary to investigate the effect of additional better-characterised tools to inhibit SMO, such as cyclopamine or other small molecule inhibitor or siRNA, on the GABAergic switch or KCC2 levels/phosphorylation.

4- Is endogenous wild type SMO in the somatosensory cortex or in the primary neutrons acting in a Hh-dependent manner? Can DN-SMO be mimicked by expressing a DN-Ptch1 lacking an extracellular loop necessary for binding to Shh? Can the GABAergic polarity switch be elicited by addition of Shh?

Minor comment:
Description of Patched/Smo as a Hh co-receptor complex is inaccurate, given the breadth of information that there is no physical interaction between them, their distinct localisation, and the known substochiometric repression of Smo by Ptch1 acting as a cholesterol transporter. Ptch1 is the Hh receptor Gas1, Boc and Cdo are interchangeable co-receptors. SMO is better described as a signal transducer or mediator, even if it is a member of the GPCR superfamily.

First revision

Author response to reviewers' comments

Dear Editor,

We are grateful for the favorable notification of our manuscript entitled "Smoothened receptor Signaling regulates the developmental shift of GABA polarity in rat somatosensory cortex" (JCS 247700).
We want to thank the reviewers for their useful comments. The revised manuscript has been amended according to their suggestions.

- According to the comments of the referee #1, we performed additional experiments to demonstrate that *in utero* electroporation using Smo-related constructs do not alter the density and localization of electroporated neuronal cells at embryonic day 20 (Figure 1A and B of the revised manuscript).
- We added new experiments on primary neuronal cell cultures with the Smo SA0-5 construct as a complementary control. Our results gained with the Smo SA0-5 are comparable to those obtained with GFP-control in qRT-PCR experiments and electrophysiological recordings (Figure 5A-C of the revised manuscript).
- According to the comments of the referee #2 we have carried out a new set of experiments to investigate whether the hyperpolarizing shift of GABA observed in Smo-CA rats might involve the target gene Gli1, downstream of Smo receptor. For this purpose, we have used the Gli1-selective blocker, GANT61, on primary neuronal cell cultures in control and Smo-CA conditions. We found that blockade of Gli1, in Smo-CA electroporated cells reversed the GABA hyperpolarizing shift (Figure 5A-C of the revised manuscript). These data suggest that Smo-CA acts through the canonical Smo-Gli1 pathway.

This manuscript has certainly benefited from the insightful revision suggestions.

© 2020. Published by The Company of Biologists under the terms of the Creative Commons Attribution License (https://creativecommons.org/licenses/by/4.0/).

We hope very much that our revised manuscript with meet the requirements for acceptance and publication in Journal of Cell Science.
Appended to this letter is our point-by-point response to the comments raised by the reviewers. Please find enclosed the revised version of the manuscript and the corresponding re-built figures.
Thank you very much for your kind consideration,

Sincerely yours,

Reviewer 1 Advance Summary and Potential Significance to Field...

In this manuscript the authors describe the consequence of electroporation of activated and inhibitory forms of Smo on the maturation of GABA neurons. It appears that Smo activation results in an earlier appearance of more mature forms of GABA neurons.

Reviewer 1 Comments for the Author...

We would like to thank the reviewer for their insightful comments and helpful suggestions.

In this manuscript the authors describe the consequence of electroporation of activated and inhibitory forms of Smo on the maturation of GABA neurons. It appears that Smo activation results in an earlier appearance of more mature forms of GABA neurons.
The delivery of constructs via electroporation is convincing and has the advantage of reaching predominantly cells lining the ventricles thus limiting delivery to fewer cell types.

1. main concern is the absence of a very obvious control, the use of Wt Smo. This is critical as the high level of expression that is sometimes achieved by electroporation using constructs with strong promoters leads to maturation and localization issues, that should be controlled for using Wt Smo.

We understand the reviewer's concern but overall, our results showed that *in utero* electroporation (IUE) of Smo-CA or Smo-DN does not reveal any differences in density or positioning of pyramidal cells when compared to control-GFP at P15. Adding new IUE experiments by the use of a complementary control with a Smo-WT construct will introduce a long delay. In an attempt to answer the question, we now investigated whether IUE of Smo- related constructs might produce alterations during the embryonic stage. We performed a complementary morphological analysis of cortical electroporated tissues at embryonic day 20 (E20). As illustrated now in modified Figure 1 (Fig. 1A and B), we found that the somatosensory cortex electroporated with Smo-CA or Smo-DN did not show any difference compared to control-GFP. These results suggest that IUE of Smo-related constructs have no gross impact at the cellular level on maturation and localization of electroporated cells.

The text has been amended accordingly in the methods (P22, L584-588) and results sections (P5, L105-L117): "Because Shh signaling is involved in cell division and growth of cortical progenitors (Araújo et al., 2014; Radonjić et al., 2016), we first investigated whether Smo-related constructs might produce alterations in the localization of electroporated neuronal cells in E20 rat embryos. We found no significant differences in the distribution of electroporated cells between Smo-CA and Smo-ΔN when compared to control-GFP (Fig. 1A and B), within the cortical plate (CP) (mean values: 92.28 %, 90.79 % and 93.34 % respectively; $p = 0.37$ for Smo-CA and $p = 0.26$ for Smo- ΔN, Mann-Whitney test), the intermediate zone (IZ) (6.10 %, 7.71 % and 5.62 % respectively; $p = 0.74$ for Smo-CA and $p = 0.21$ for Smo-ΔN, Mann-Whitney test) and the subventricular/ventricular zones (SVZ/VZ) (1.61 %, 1.49 % and 1.04 % respectively; $p = 0.58$ for Smo-CA and $p = 1.49$ for Smo-ΔN, Mann-Whitney test).
These findings suggest that electroporation of Smo-related constructs have no gross impact at the cellular level on maturation and localization of electroporated cells in developing rat somatosensory cortex."

To extent the validation of the Smo-related constructs, we performed new electrophysiological recordings and single cell qRT-PCR experiments on primary neuronal cell cultures with the Smo SA0-5 as a complementary control. This construct mimic the overexpression of Smo related-constructs without overactivation of its signaling pathway by potential endogenous agonists (Chen et al., 2011).

© 2020. Published by The Company of Biologists under the terms of the Creative Commons Attribution License (https://creativecommons.org/licenses/by/4.0/).

Our results revealed that Gli1 mRNA levels or GABA reversal potential (EGABA) were similar between Smo SA0-5 and control- GFP in electroporated cells (see Fig. 5 A-C of the revised manuscript). The text has been amended accordingly in the methods (P23, L597-601; P26, L687-688) and results sections (P9, L224-L232):

"To ensure that the overexpression of Smo-related constructs did not modify E_{GABA} by itself, we compared the values between Smo SA0-5, a constitutively inactive form of Smo (Chen et al., 2011), and control mCherry in transfected neurons. We found that the E_{GABA} values were identical between Smo SA0-5 and control mCherry (median values: -56.68 mV in Smo SA0-5 vs -53.16 mV in mCherry; $p = 0.50$, Mann-Whitney test; Fig. 5B). Consistent with our results obtained in somatosensory cortical slices, the measurements of E_{GABA} in Smo-CA transfected neurons showed more hyperpolarized values when compared to control mCherry and Smo SA0-5 (-61.9 mV in Smo-CA vs -53.16 mV in control and -56.68 mV in Smo SA0-5; $p = 0.0004$ and $p = 0.02$ respectively, Mann-Whitney test; Fig. 5A and B)."

We hope that these complementary experiments will convince the referee that the GFP plasmid is a solid control in our IUE experiments.

2. The demonstration that the transcriptional Hh pathway is affected using Gli and Ptch1 RNA levels is not very convincing and might not demonstrate the barely significant difference when compared to this appropriate control.

We thank the reviewer for this comment. Our qRT-PCR analysis was carried out on mRNA extracted from electroporated cortices. As illustrated in Fig. 1 and 2, the electroporation of various plasmids was expressed in a limited number of cells (i.e. pyramidal cells in layers 5/6), which probably explain the small but significant difference in Gli1 and PTCH1 mRNAs expression between Smo-CA and Smo-ΔN. In order to consolidate these results gained in electroporated tissues, we performed new experiments with single cell qRT-PCR on transfected neurons with the Smo-related constructs (GFP, Smo-CA, Smo-ΔN and Smo SA0- 5). We found that the expression levels of Gli1 in Smo-CA condition were significantly higher when compared to controls (GPF and Smo SA0-5) and conversely decreased in Smo- ΔN conditions (Fig. 5C). These additional data on primary neuron culture confirm our results obtained in cortical electroporated tissues.
Fig. 5 has been modified accordingly. We also adapted the text in the methods (P23, L597-601) and results sections accordingly (P10, L241-252):

"Next, we quantified by single cell qRT-PCR the Gli1 mRNA levels from Smo-transfected neurons, with or without the application of 10 µM GANT61. In Smo-CA transfected cells, Gli1 mRNA levels were significantly increased when compared to control and Smo SA0-5 conditions (0.01 a.u. in Smo-CA vs 0.0029 a.u. in control and 0.0036 a.u. in Smo SA0-5; $p = 0.008$ and $p = 0.008$ respectively, Mann- Whitney test; Fig. 5C) and conversely downregulated in Smo-ΔN neurons (0.0004 a.u. in Smo-ΔN vs 0.0029 a.u. in control and 0.0036 a.u. in Smo SA0-5; $p = 0.015$ and $p = 0.016$ respectively, Mann- Whitney test; Fig. 5C). Moreover, the effect of Smo-CA on the target gene Gli1 was blocked in the presence of GANT61 (0.0024 a.u. in Smo CA + GANT61; $p = 0.016$ when compared to Smo-CA alone; $p = 0.41$ and $p > 0.99$ when compared with control mCherry and Smo SA0-5 respectively, Mann-Whitney test; Fig. 5C). These results, therefore, suggest that the constitutively active form of Smo prematurely shifts GABA polarity through the activation of the canonical Gli-dependent transcription pathway."

3. Given the marginal induction/repression of the Hh response pathway after electroporation, a further demonstration of the involvement of the transcription Hh response pathway would be the use of blocking and activated forms of Gli. In particular the truncated forms of Gli3 are much more powerful dominant negatives that the inhibitory forms of Smo.

We thank the reviewer for this comment. We performed complementary gramicidin and qRT- PCR (see response to Q2) experiments to further elucidate the Shh-Smo downstream pathway by the use of GANT61, a downstream inhibitor of Shh-Smo canonical signaling (Lauth et al., 2007). Our results showed that treatment with 10 µM GANT61 for 48h led to a significant reduction of the increased expression levels of the Shh-Smo target gene Gli1 in Smo-CA transfected neurons. We included these results in Fig. 5C and in the result section (P10, L235- 240):

© 2020. Published by The Company of Biologists under the terms of the Creative Commons Attribution License (https://creativecommons.org/licenses/by/4.0/).

“To further investigated whether Smo is acting through the canonical Gli-dependent pathway, we used the Gli antagonist, GANT61, a downstream inhibitor of Shh-Smo canonical signaling pathway (Lauth et al., 2007). We found that the application of GANT61 abolished the hyperpolarized E_{GABA} values observed in Smo-CA expressing neurons by restoring it to values similar to control (-54.92 mV in Smo-CA + GANT61 vs -61.9 mV for Smo-CA and -53.16 mV in control; respectively p = 0.04 and p = 0.36, Mann-Whitney test; Fig. 5A and B).”

Although mentioned several times, nowhere in this paper is it demonstrated that the effects on GABA maturation are mediated by Shh, only that a possible upregulation of the Hh response downstream of Smo has an effect on GABA neuron maturation. For that, something like 5E1 blocking experiments have to be performed. It would be a good addition to the paper to demonstrate that Shh electroporation in general gives the same phenotype as electroporation of activated Smo, and it would help to demonstrate that Shh is involved the GABA neuron maturation.

We agree with the comment of the reviewer that the present study did not focus on the effect of Shh signaling on GABA maturation. Indeed, we have investigated the effect of Smo and not Shh and we apologize for this misleading phrasing. We now refer to the Smo or Shh-Smo pathway instead of Shh throughout the manuscript. Furthermore, the acute action of Shh or Smo on the maturation of GABAergic networks has been recently published by our team:

Delmotte et al., 2020 (Frontiers in Cellular Neuroscience 2020; 14: 98). In this manuscript we showed that application of Smo agonist (SAG) induced a significant increase in Gli1 mRNAs whereas acute treatment with SAG does not change Gli1 expression levels. As suggested, we added new gramicidin experiments showing that the application of the Gli1 inhibitor GANT61 completely abolished the GABA hyperpolarizing shift in Smo-CA expressing neurons and restored EGABA to values similar to controls (Fig. 5A and B).

Altogether, these results suggest that Smo-CA signaling prematurely shifts GABA polarity via the canonical activation of Gli1.

Moreover, in the present study, we sought to focus on the role of Smo signaling on the GABA polarity shift and KCC2 regulation in the early postnatal period through its chronic activation or inhibition. However, whether acute or chronic manipulation of Shh regulates GABA polarity and KCC2 cell surface stability remains an interesting question.

Reviewer 2 Advance Summary and Potential Significance to Field...

The study of Delmotte et al. elegantly shows a role of the Hedgehog core transducer Smoothened (SMO) in maturation of the GABAergic properties in the somatosensory cortex in rats, which is essential to prevent seizures in adult animals. It shows a novel function of SMO in promoting the stabilisation of the K^+/Cl^- KCC2 channel at the plasma membrane, to mediate a developmental shift of GABAa receptors form depolarising to hyperpolarising.

Reviewer 2 Comments for the Author...

The study combines in utero electroporation of rat embryos to introduce constitutively active or dominant negative SMO; together with expression in primary neuron cultures to show that SMO promotes stabilisation of the K^+/Cl^- KCC2 channel at the plasma membrane, to mediate a developmental shift of GABAa receptors form depolarising to hyperpolarising. These findings are convincing and well-controlled, however they generate key questions on the specific function of SMO in the process and the role of Hh ligands in controlling SMO's activity:

1. Is SMO acting through the canonical (GLI-dependent) Hh pathway? Experiments in neuronal cultures could be performed using GLI inhibitors and/or GLI3R to investigate their involvement.

We thank the reviewer for this comment. We have now added gramicidin experiments on Smo SA0-5 and Smo-CA expressing neurons in the presence/absence of GANT61 (10 μM) during 48h before

© 2020. Published by The Company of Biologists under the terms of the Creative Commons Attribution License (https://creativecommons.org/licenses/by/4.0/).

recordings (see responses to Q2 and Q3 Ref#1). We show that GANT-61 completely recovered EGABA for Smo-CA neurons to values similar to control conditions (Fig. 5A and B).
These results suggest that Smo-CA signaling prematurely shifts GABA polarity via the canonical activation of Gli1. The values with GANT61 experiments have been added in Fig. 5 and in the results sections (P10, L235-(240).

2. a previous report from one of the authors revealed a non-canonical role of SMO in neuronal progenitors Ca++ spikes, through coupling to G inhibitory proteins. Are Gi proteins mediators of SMO in this context?

We thank the reviewer for this comment. G_{ai} have, indeed, been previously demonstrated to be involved in canonical and non-canonical Smo signaling in various cell-type models. Additionally, Ca^{2+} levels have been involved in Shh signaling in a wide variety of models.
As the reviewer highlighted, we previously demonstrated that a non-canonical Shh signaling acutely modulates Ca^{2+}-mediated spontaneous electrical activity in the embryonic spinal cord through a G_{ai} protein (Belgacem and Borodinsky, 2011). However, in this context, we showed that this G_{ai}-dependent non-canonical signaling acts as a negative regulator of the canonical Shh signaling. Indeed, activation of Smo leads to an increase of Ca^{2+} spike frequency, leading to PKA activation and subsequent inhibition of the activator forms of Gli transcription factors (Gli1 and $Gli2^{A}$) concomitant with an enhancement of the repressor forms of Gli ($Gli3^{R}$) in embryonic spinal neurons (Belgacem and Borodinsky, 2015).
In this current study, we show rather an enhancement of Gli activity upon Smo activation (Fig. 3B and 5C). Additionally, the new experiments we performed using Gli transcription factor inhibitor GANT61 suggest that Smo acts through Gli-dependent canonical signaling to modulate the reversal potential of GABA (Fig. 5C).
Altogether, this body of data suggests that, in this model, the non-canonical Smo- signaling pathway we previously described (Belgacem and Borodinsky, 2011, 2015) is not involved. This highlights the diversity of signaling pathways recruited by Smo among cell types, central nervous structures and developmental stages. It will be therefore of great interest to further explore, in future studies, the precise Smo signaling pathway involved in our model and, particularly, if G_{ai} protein and Ca^{2+} dynamics are part of this mechanism.

We added the following sentence in the discussion (P18, L460-474):
"This effect of Smo was blocked by preincubation with the Gli transcription factor inhibitor GANT61, suggesting that in the current study, Smo acts through Gli-dependent canonical signaling (Belgacem et al., 2016) to modulate the reversal potential of GABA. Our recent research demonstrated that Smo also participates to the maturation of GABAergic networks in the postnatal rat hippocampus through a non-canonical pathway (Delmotte et al., 2020). A non-canonical Shh signaling has been shown to acutely modulates Ca^{2+}-mediated spontaneous electrical activity in the embryonic spinal cord through a G_{ai} protein (Belgacem and Borodinsky, 2011). In this context, the G_{ai}-dependent non-canonical signaling acts as a negative regulator of the canonical Shh signaling. The activation of Smo leads to an increase of Ca^{2+} spike frequency, leading to PKA (protein kinase A) activation and subsequent inhibition of the activator forms of Gli transcription factors (Gli1 and $Gli2^{A}$) concomitant with an enhancement of the repressor forms of Gli ($Gli3^{R}$) in embryonic spinal neurons (Belgacem and Borodinsky, 2015). Here, we show rather an enhancement of Gli activity upon Smo activation, indicating that the non-canonical Smo-signaling pathway is not involved (Belgacem and Borodinsky, 2011; Belgacem and Borodinsky, 2015). This highlights the diversity of signaling pathways recruited by Shh signal transducer Smo among cell types, central nervous structures, and developmental stages."

3. the inactive form of SMO used, called dominant-negative, is a deletion of a portion of the C-tail that has showed inability to activate GLI-dependent transcription and reduces endogenous SMO activity, therefore being proposed as a dominant negative. However, the inhibitory activity of this DN-SMO mutant is far from complete, and its effect on non-canonical HH signalling, in particular on Gi protein activation is unknown. For example, wild type SMO does not require the C-tail for Gi coupling, but the effect of this partial deletion is unknown. Therefore, it will be necessary to investigate the effect of additional better-characterised tools to inhibit SMO, such as cyclopamine or other small molecule inhibitor or siRNA, on the GABAergic switch or KCC2 levels/phosphorylation.

© 2020. Published by The Company of Biologists under the terms of the Creative Commons Attribution License (https://creativecommons.org/licenses/by/4.0/).

We thank the reviewer for this comment and agree that Smo Δ570-581 (Kim et al. 2009) is not a dominant-negative form of Smo, *stricto sensu,* since it does not fully inhibit wild-type Smo activity and that its mechanism of action is not fully understood. It is however interesting to note that Smo Δ570-581 overexpression reduces Gli1 and Ptch1 mRNA levels in rats sommatosensory cortex (Fig 3B and C), indicating, at least, a partial inhibitory effect on endogenous Smo signaling. In addition, while all cells are not electroporated (Fig. 2A), mRNA levels have been measured on whole somatosensory cortex tissues, including non- electroporated cells. The decrease of Gli1 and Ptch1 mRNA levels observed under these conditions suggests that, in this model, Smo Δ570-581 overexpression behaves similarly as a dominant-negative Smo on Gli activity.
Accordingly, we changed the term “dominant negative” by “negative phenotype” throughout the manuscript.
In order to better know the mechanisms involved in this process, it will be of great interest in a future study, to block endogenous Smo-dependent signaling using cyclopamine or siRNAs as suggested by Reviewer#2 and assess the effects on the GABAergic polarity switch and/or KCC2 trafficking.

4. Is endogenous wild type SMO in the somatosensory cortex or in the primary neutrons acting in a Hh-dependent manner? Can DN-SMO be mimicked by expressing a DN- Ptch1 lacking an extracellular loop necessary for binding to Shh? Can the GABAergic polarity switch be elicited by addition of Shh?

In this study, we decided to focus on the consequences of Smo modulation on GABAergic transmission maturation in the somatosensory cortex. We agree with the reviewer that the next step will be to understand the role of endogenous Shh, that is present in the somatosensory cortex during the perinatal period, in this process and it will be the subject of a future study. The acute action of Shh-Smo signaling on the maturation of GABAergic networks has been recently published by our team: Delmotte et al., 2020 (Frontiers in Cellular Neuroscience 2020; 14: 98). In this paper, we showed that application of Smo agonist (SAG) increases the frequency of the synchronized electrical activity called Giant Depolarizing Potentials (GDP), which requires a depolarizing action of GABA for their initiation, and also enhances spontaneous GABA post-synaptic currents in the rodent hippocampus during the early postnatal period.

Minor comment:
Description of Patched/Smo as a Hh co-receptor complex is inaccurate, given the breadth of information that there is no physical interaction between them, their distinct localisation, and the known substochiometric repression of Smo by Ptch1 acting as a cholesterol transporter. Ptch1 is the Hh receptor, Gas1, Boc and Cdo are interchangeable co-receptors. SMO is better described as a signal transducer or mediator, even if it is a member of the GPCR superfamily.

We thank the reviewer for this clarification and agree with it. While Smo is often described as a Shh co-receptor, it is not true as Shh molecule does not bind on Smo. We will correct the text accordingly and describe Smo as a “Shh signal transducer” in lieu of a “Shh co-receptor”.

<u>Second decision letter</u>

MS ID#: JOCES/2020/247700

MS TITLE: Smoothened receptor signaling regulates the developmental shift of GABA polarity in rat somatosensory cortex

AUTHORS: Quentin Delmotte, Mira Hamze, Igor Medina, Emmanuelle Buhler, Jinwei Zhang, Yesser H Belgacem, and Christophe Porcher
ARTICLE TYPE: Research Article

I am happy to tell you that your manuscript has been accepted for publication in Journal of Cell Science, pending standard ethics checks.

© 2020. Published by The Company of Biologists under the terms of the Creative Commons Attribution License (https://creativecommons.org/licenses/by/4.0/).

Reviewer 1

Advance summary and potential significance to field

In this manuscript the authors describe the consequence of electroporation of activated and inhibitory forms of Smo on the maturation of GABA neurons. It appears that Smo activation results in an earlier appearance of more mature forms of GABA neurons.

Comments for the author

I am disappointed that none of the electroporation that would enhance the paper were performed. I understand that this a significant amount of work, likely complicated by the pandemic. The single cell analysis is a significant improvement of this manuscript.

Reviewer 2

Advance summary and potential significance to field

The revised manuscript is much improved and will be a nice addition to the literature of novel functions of the Hh pathway.

Comments for the author

The revised manuscript is much improved and will be a nice addition to the literature of novel functions of the Hh pathway.

© 2020. Published by The Company of Biologists under the terms of the Creative Commons Attribution License (https://creativecommons.org/licenses/by/4.0/).
